# Shifts in Bacterial Communities of Eggshells and Antimicrobial Activities in Eggs during Incubation in a Ground-Nesting Passerine

**DOI:** 10.1371/journal.pone.0121716

**Published:** 2015-04-16

**Authors:** Stéphanie Grizard, Maaike A. Versteegh, Henry K. Ndithia, Joana F. Salles, B. Irene Tieleman

**Affiliations:** 1 Animal Ecology Group, Centre for Ecological and Evolutionary Studies, University of Groningen, Groningen, The Netherlands; 2 Department of Microbial Ecology, Centre for Ecological and Evolutionary Studies, University of Groningen, Groningen, The Netherlands; 3 Department of Zoology, Ornithology section, National Museums of Kenya, Nairobi, Kenya; University of Akron, UNITED STATES

## Abstract

Microbial invasion of egg contents is a cause of embryonic death. To counter infection risks, the embryo is protected physically by the eggshell and chemically by antimicrobial proteins. If microbial pressure drives embryo mortality, then females may have evolved, through natural selection, to adapt their immune investment into eggs. Although frequently hypothesized, this match between immune allocation and microorganisms has not been explored yet. To examine if correlations between microbes on eggs and immunity in eggs exist, we collected eggs from red-capped larks (*Calandrella cinerea*) and simultaneously examined their bacterial communities and antimicrobial components—pH, lysozyme and ovotransferrin—during natural incubation. Using molecular techniques, we find that bacterial communities are highly dynamic: bacterial abundance increases from the onset to late incubation, Shannon’s α-diversity index increases during early incubation stages, and β-diversity analysis shows that communities from 1 day-old clutches are phylogenetically more similar to each other than the older ones. Regarding the antimicrobials, we notice a decrease of pH and lysozyme concentration, while ovotransferrin concentration increases during incubation. Interestingly, we show that two eggs of the same clutch share equivalent immune protection, independent of clutch age. Lastly, our results provide limited evidence of significant correlation between antimicrobial compounds and bacterial communities. Our study examined simultaneously, for the first time in a wild bird, the dynamics of bacterial communities present on eggshells and of albumen-associated antimicrobial components during incubation and investigated their relationship. However, the link between microorganisms and immunity of eggs remains to be elucidated further. Identifying invading microbes and their roles in embryo mortality, as well as understanding the role of the eggshell microbiome, might be key to better understand avian strategies of immune maternal investment.

## Introduction

During embryonic development, microbial infection of egg contents may be a cause of death and ultimately hatching failure [1–5]. From the time an egg is laid, prior to the onset of incubation [6], and during the entire incubation, the embryo is threatened by microbial invasions that might affect its viability [2, 7]. To minimize invasions, eggs possess physical and chemical barriers including the shell, cuticle, membranes [8–10], and the albumen. Regarding the latter, its fibrous and viscous nature [11] as well as its antimicrobial defences, guaranteed by bactericidal and bacteriostatic protein activities, represent a crucial shield against microbes that the embryo may face [12–14]. The transmission of antimicrobials to the albumen is one of the parental strategies to confer protection to the future chick. Considering that the amount deposited at the time of laying cannot be further adjusted and should efficiently protect the embryo until hatching [15, 16], the level of immune defences that females invest into the albumen must have evolved, through natural selection, to optimize protection from the risk of trans-shell microbial penetration [15–17].

The risk of the trans-shell penetration comes from the particular microbes present on the eggshell surface that might have the ability to pass through shell pores and then invade egg contents. Interestingly, the microbial communities associated with eggshells are thought to be modified by incubation which reduces or limits bacterial growth [1–3] by maintaining shell dryness [18, 19] and controls bacterial richness [20], as observed in experimental studies comparing incubated and exposed eggs (but see [21, 22]). The few studies investigating microbial dynamics during incubation focused on two or three incubation time points, and provided contrasting results. Studies performed in pied flycatchers (*Ficedula hypoleuca*) [23] and in pearly-eyed thrashers (*Magarops fuscatus*) [20] found that eggshell bacterial morphological types, and assemblage composition and abundance, respectively, remained fairly constant over incubation. In contrast, four studies showed that bacterial communities on eggshells are not static: a decreased diversity and an increased abundance between early and late incubation days were observed in pigeons (*Columba livia*) [24] and magpies (*Pica pica*) [22]. An increased abundance was also observed on mallards (*Anas platyrhynchos*) [21] whereas changes in community structure were observed in house wrens (*Troglodytes aedon*) [25]. Although a full depiction of the eggshell microbiome is necessary to shed light into their potential role in egg invasion, its dynamics over a continuum of incubation time points from laying to late incubation stages remain unexplored.

As incubation begins, albumen goes through physical and chemical modifications, including alterations of the antimicrobial functions. In domestic white leghorn (*Gallus gallus*), fluctuation in pH has been well-described [26] together with its bactericidal role and stimulating effect on antimicrobials [27]. Changes in activities of two major antimicrobial proteins, lysozyme and ovotransferrin, were also reported. Over the entire incubation, Cunningham found a decrease in lysozyme and ovotransferrin activities [28]. Focusing on early incubation stages, Fang *et al*. noted a decrease followed by an increase of lysing activity as well as an increased iron-binding activity of ovotransferrin [29]. In parallel, studies on wild birds have instead evaluated factors responsible for the albumen antimicrobial allocation among eggs, clutches or species. For instance, lysozyme concentration was shown to decrease with laying order—in barn swallows (*Hirundo rustica*) [30], red-legged partridges (*Alectoris rufa*) [31], and grey partridges (*Perdix perdix*) [32] (but see [33])—in line with the hypothesis that longer exposure to ambient conditions increases microbial invasion rates [1]. Despite the effort in describing antimicrobial deposition, how their activities vary during incubation remains unknown in wild birds. Importantly, simultaneous analyses of the dynamics of both the level of antimicrobial compounds and the eggshell microbiome are currently lacking.

To assess the dynamics of microbes and antimicrobials in eggs, we studied the free-living red-capped lark (*Calandrella cinerea*), an open-cup ground-nester breeding in the tropics. Eggs in open-cup nests may suffer from higher microbial growth than those in cavity nests [34] (but see [35]), therefore enhancing the probability of detecting relationships between microbes and antimicrobials. Moreover, previous work on adult larks across climates showed strong associations between immune plasma indices and microbial density faced by adults [36], highlighting the potential effect of microbes as selective forces impacting immune defences.

In order to obtain a dynamic view of bacterial communities associated with eggshells, and of albumen antimicrobials, we collected red-capped lark eggs through the whole incubation period. We hypothesized that antimicrobial compounds should ensure an effective protection against microbial trans-shell invasions until hatching. Therefore, we first examine the dynamics of eggshell bacterial communities by investigating their structure, abundance and composition, using molecular tools. Next, we describe fluctuations of pH, lysozyme and ovotransferrin concentrations. In addition, as females typically lay two eggs per clutch, we investigate if both eggs possess similar level of immune defences, independent of clutch age. Lastly, we examine if particular features of eggshell bacterial communities correlated with antimicrobials, to determine their potential covariation during incubation.

## Materials and Methods

### Ethics statement

The National Museums of Kenya (NMK) is a quasi-government institution with the mandate to carry out scientific research, and our research was part of the fulfillment of its mandate. The NMK takes the position of an Institutional Animal Care and Use Committee, and Government Authority, because it is the single institutional authority on matters regarding to birds in Kenya. The NMK is the only responsible for taking decisions about ethical consideration upon our work. Our overall study and sampling procedures were approved by the NMK, but specific permission was not required to work in our study location (Seminis field, Plateau of South Kinangop, 0°42’S, 36°36’E). Seminis is a public land from which public institutions like the NMK have access to. We notified local government authorities (local Chief and local District Officer) of our activities and worked together with the local community (Friends of Kinangop Plateau) as field assistants. The local authorities were informed about our work as we worked in their area of jurisdiction but were not responsible for delivering permission. The NMK is a registered center for CITES (Convention of International Trade in Endangered Species of Wild Fauna and Flora) (CITES registration No.001). Our study species, the red-capped lark *(Calandrella cinerea)*, was approved as not appearing in the list of endangered or protected species (http://www.iucnredlist.org/details/22717319/0).

### Study area and bird species

Our study took place in the open highland grassland field of Seminis, on the Plateau of South Kinangop, Kenya (0°42’S, 36°36’E; 2556m amsl). The site is characterized by high annual precipitation (over 1000mm/year; daily average: 2.9mm (±0.26); range: 0.0–35.2mm) and tropical temperatures (daily average range: minimum temperature 5.5°C (±0.11)—maximum temperature 24.2°C (±0.29)) obtained by our own daily weather data recording at the site during the complete year 2012 (S1 Appendix). In Seminis, red-capped larks (*Calandrella cinerea*) mostly breed at the onset of rains. Females typically lay one egg per day and two eggs per clutch in a shallow open-cup nest lined with grasses and/or rootlets. Incubation is initiated the first day of clutch completion (hereafter ‘day 1’) and eggs hatch synchronously 12 days later [37].

### Egg sampling and processing

To follow nest construction and egg laying, we monitored breeding activity daily. When possible, we discreetly marked the first laid eggs with an indelible dot. We collected the two eggs per nest (i.e. the complete clutch), and did so at different time points, ranging from day 1 to day 11 after clutch completion, and mostly during the five first days (82.6%). We collected six eggs from 16 to 30 January 2012 and forty-six from 14 March to 25 April 2012. S2 Appendix describes the egg/nest sample size per clutch age.

We collected and handled eggs wearing gloves sterilized with 70% ethanol. Eggs were individually stored in sterile bags (Whirl-Pack Write-On Bags, Nasco, Fort Atkinson, WI), kept on ice during fieldwork (max: 7h), then frozen at -20°C. In the field station, we performed egg dissections following Grizard *et al*. [18] and kept parts at -20°C. To assess egg age when laying date was unknown, we looked at yolk shape (round/oblong) for the youngest eggs (from day 1 to 4 after clutch completion), and we examined the embryonic dimensions (body length and width, and head length) and the presence/absence and amount of down feathers for older eggs (from day 5 to 11 after clutch completion) [38, 39]. All samples were transferred to the Netherlands in frozen thermos bottles, and then stored again at -20°C immediately upon arrival. All molecular work and antimicrobial assays were carried out in the Netherlands.

### Assessing bacterial communities in eggshells

We extracted and quantified microbial DNA from forty-six eggshells following Grizard *et al*. [24]. Briefly, after crushing the entire eggshells into liquid nitrogen, we extracted DNA from the eggshell powder using the Fast DNA SPIN kit (MP Biomedicals LLC, Solon, OH). We followed this ‘crush’ protocol except that the final elution step was done in a final volume of 150 μL. DNA concentration was determined by fluorescent quantification using Quant-iT PicoGreen dsDNA kit (Molecular Probes Inc., Eugene, OR) [24]. The extracted DNA was further used as template to determine the abundance and diversity of bacterial communities. Due to the often low concentration of extracted DNA per sample, not all samples could be analyzed for both bacterial abundance and diversity, explaining differences in sample size per method (S1A Table, S2 Appendix).

We determined the bacterial abundance by quantitative PCR targeting partial region of the 16S rRNA gene using the primer set FP16S/RP16S. The efficiency of the reaction was 102.0% (±1.46) and we carried out quantifications using variable amount of DNA template (3.1ng (±0.50)). Details about the overall procedure are described in Grizard *et al*. [24]. We calculated abundances per g of eggshell, after correction for the amount of DNA template per sample, and obtained log copy number of the 16S rRNA gene for twenty-nine eggshells (S1A Table, S2 Appendix).

We assessed bacterial communities by 454-Roche multitag pyrosequencing of the V4-V6 region of the 16S rRNA gene, using the primer set 16s-515F (5’-TGYCAGCMGCCGCGGTA-3’) and 16s-1061R (5’-TCACGRCACGAGCTGACG-3’), where each set was coupled with a unique barcode (MID Roche) per sample. We carried out reaction in 25μL containing 1.25U FastStart High Fidelity Enzyme (Roche Applied Science, Mannheim, Germany), 1x Reaction Buffer without MgCl_2_, 2.3mM MgCl_2_ stock solution, 0.20mM PCR nucleotide mix, 0.50mg/ml Bovine Serum Albumin (Roche Applied Science), 0.20μM primer/barcode and 1ng DNA template. The thermal cycle started with 5min at 95°C, followed by 35 cycles at 95°C for 40s, 56°C for 45s, 72°C for 40s, and ended with 10min at 72°C. We ran samples at least in triplicate and checked PCR mixes for the absence of contamination with negative controls of UltraPure Water (Invitrogen, Carlsbad, CA). All samples were consistently amplified. We pooled amplicons together to minimize PCR bias, and slowly ran them in a 2.5% (w/v) agarose gel to check their size and integrity. We excised and purified bands with the QIAquick Gel Extraction kit (Qiagen, Hilden, Germany). We pooled purified amplicons from the same sample together and dried them in a vacuum concentrator at 30°C (Concentrator 5301, Eppendorf, The Netherlands). We measured their concentrations by fluorescence using Quant-iT PicoGreen dsDNA kit (Molecular Probes Inc., Eugene, OR). Purified amplicons from twenty-seven samples were pooled in equimolar concentrations and ran on a Roche GS-FLX 454 automated pyrosequencer (Titanium chemistry) at Macrogen (Korea).

We processed the pyrosequencing raw data using the Quantitative Insights Into Microbial Ecology (QIIME) toolkit (version 1.7.0) [40]. We trimmed sequences for quality by assigning them into Operational Taxonomic Units (OTUs) at 97% nucleotide identity, using ‘close reference’ function and ‘Greengenes’ reference database (http://greengenes.lbl.gov/). Only sequences matching the database were considered for analyses [41]. After quality trimming, 25,503 sequences from the twenty-seven samples were retrieved (S2 Appendix). We built OTUs using UCLUST [42]. One representative sequence per OTU was selected and aligned against ‘Greengenes’ using PyNAST [43] and later taxonomically classified using RDP classifier [44].

We rarefied the number of sequences to 160 per sample to minimize effects of sampling effort upon α-diversity metrics. In this process, seven samples were discarded, reducing our overall sample size to twenty eggshells (S1A Table, S2 Appendix). The cut-off we applied ensured a good coverage of the OTU diversity (95.7% (±0.48); range: 88.9%-97.8%)). From these twenty samples, we calculated the following α-diversity metrics: OTU richness (equivalent to species richness), Chao1 index (estimated species richness), Shannon’s diversity index (based on OTU richness and evenness) and Faith’s phylogenetic diversity index (phylogenetic relationship between OTUs). β-diversity analyses among eggshells were performed using weighted and unweighted UniFrac distance matrices [45] and Principal Coordinates Analysis (PCoA). Bacterial communities were discriminated based on the three first axes of the PCoA plots and the percentage of variability reported per axis. All α- and β-diversity metrics were generated using QIIME.

We constructed phylogenetic trees (S3 Fig) by filtering each OTU (one representative sequence) alignment using the ‘Lanemask’ template file to remove common gaps and by manually assigning each OTU using RDP classifier (http://rdp.cme.msu.edu/index.jsp). All sequences were aligned with ClustalW in MEGA 5.2 software [46]. We generated, explored, and visualized trees using MEGA 5.2. We implemented OTU tables obtained from QIIME in each tree using the Interactive Tree Of Life, online tool [47].

### Antimicrobial assays

We recorded albumen pH using a digital pH meter (model 60, Jenco Instruments, San Diego, CA) for forty-two eggs. We assessed lysozyme concentrations following Horrocks *et al*. [18] and ovotransferrin concentrations following Horrocks *et al*. [48] except that we used 10μL of albumen instead of plasma. Concentrations were measured for thirty-eight eggs (S1B Table, S2 Appendix).

For lysozyme and ovotransferrin assays, a pool of three chicken egg albumen was run within each plate to assess intra-and inter-assay variation. The intra-assay coefficients of variation were 14.0% (n = 9 plates) and 9.3% (n = 7 plates), respectively. The inter-assay coefficients of variation were 17.4% and 15.8%, respectively.

### Statistical analyses

We analyzed eggshell bacterial β-diversity based on weighted and unweighted UniFrac distance matrices. We only compared the phylogenetic similarities within communities of 1 day-old eggshells and of 5 days-old eggshells; the restricted sample size (one or two eggs) for each of the other days impeded comparisons. The phylogenetic similarity is a straightforward transformation of the phylogenetic distance into percentage; closer an eggshell was to another one, smaller was the phylogenetic distance between them, and higher was their phylogenetic similarity. We compared similarities between communities of 1 day-old and 5 days-old using Student’s T- test.

We analyzed bacterial abundance, taxonomical data, pH, lysozyme and ovotransferrin concentrations with linear mixed-effects models (package nlme [49]). We assigned nest as a random factor, as we frequently had two observations (eggs) per nest, and included laying order, clutch age, Julian day, and pH, and their two-way interactions, as fixed factors. To test the effect of laying order, we assigned the value ‘1’ to the first laid egg of a clutch and ‘2’ to the second one. When the laying order was unknown, we gave ‘1.5’ to both eggs. Including or excluding eggs with unknown laying order did not change the significance of the model outputs. We simplified models using backward elimination based on log-likelihood ratio tests and used P<0.05 as selection criterion. We tested for the normality of residuals of final models using Shapiro tests. None of them deviated from Gaussian distribution. We reported mean values of models, and other averages, with their standard error.

After checking which distribution best fitted our data, we analyzed α-diversity indices with generalized linear mixed-effects model, following gamma distribution (package lme4, [50]).

We calculated repeatability, standard error, and coefficients of variation (CV) following Versteegh *et al*. [51]. We calculated repeatability for complete nests, i.e. containing two eggs, with the equation:

repeatability = (internest variance) / (intranest variance + internest variance).

We obtained the values of internest and intranest variances from the mixed-effect model that included clutch age (significant main effect) and nest as a random factor. We tested nest effect associated with repeatability using the likelihood-ratio test and χ^2^-statistic to evaluate statistical significance. We obtained the average within-nest (CVw) by calculating the average of nest CVw’s, using the standard deviation and the mean per nest, and the average among-nest (CVa) by averaging the two values per nest, and calculating standard deviation and mean of these nest averages. We used R 2.15.3 for statistical analyses [52].

To explore the relationships between bacterial communities and antimicrobials, we included in our analyses samples from which both sequencing and antimicrobial data were available: sixteen for lysozyme and ovotransferrin/bacteria, and seventeen for pH/bacteria (S2 Appendix). We examined the correlation between taxonomical composition (dominant phyla—surrogate of Gram-positive and Gram-negative types—and classes) and α-diversity indices with each antimicrobial using linear mixed-effect models. We also determined whether the abundance of main OTUs (seventeen main OTUs—defined by their presence in at least ten samples) correlated with each antimicrobial using Pearson correlation. In QIIME, the script otu_category_significance.py, with Pearson correlation test, determined whether OTU abundance was positively or negatively correlated with a continuous variable (lysozyme, ovotransferrin, or pH).

## Results

### Bacterial communities in relation with clutch age

#### Bacterial abundance

**Fig 1:**
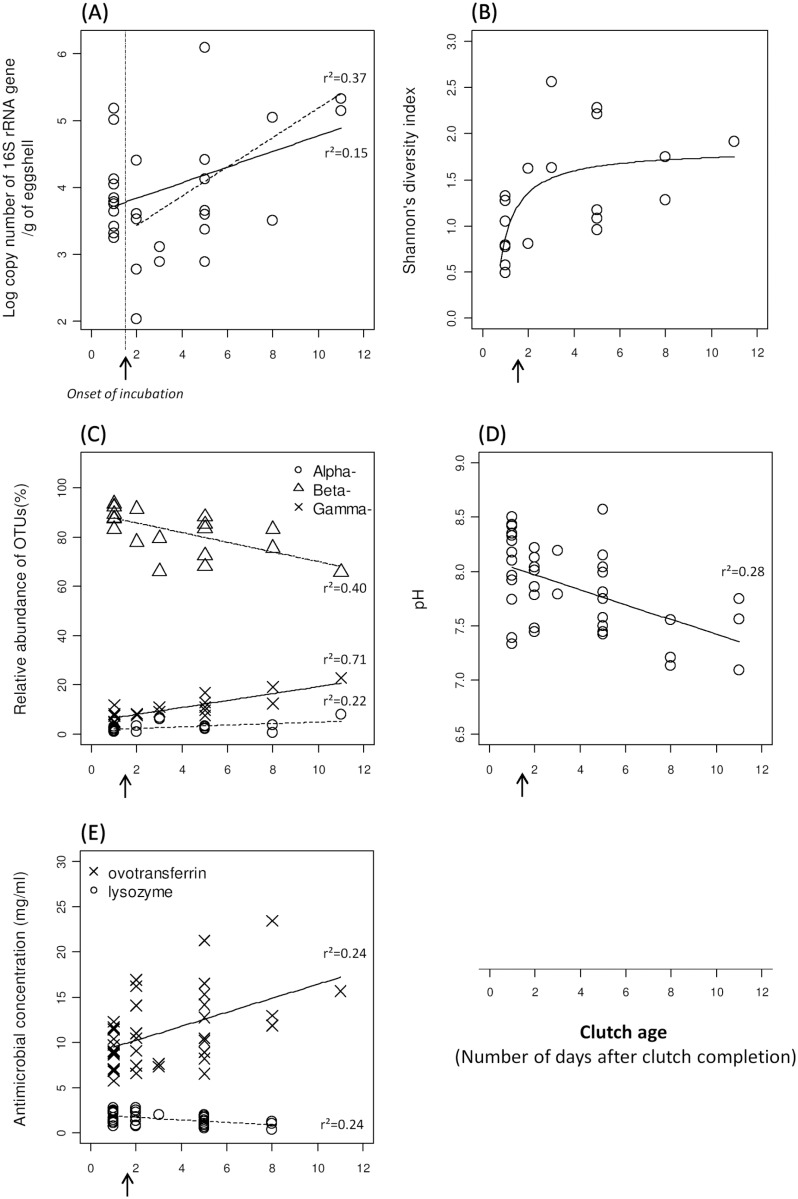
Egg-related bacteria and antimicrobials associated with clutch age. The clutch age is the number of days that the completed clutch spent in the nest; day 1 is the day of clutch completion. Incubation starts between day 1 and day 2 (*arrow*). ‘r^2^’ corresponds to the coefficient of determination between clutch age and bacterial/antimicrobial data. (A) Bacterial abundance is examined from day 1 to day 11 (*full line*) and from day 2 to day 11 (*dashed line*). (B) Shannon‘s diversity index. (C) Relative abundance of Operational Taxonomic Units (OTUs) of three main classes of *Proteobacteria*: *Alphaproteobacteria* (Alpha*-*) (*dotted line*), *Betaproteobacteria* (Beta*-*) (*dashed line*) and *Gammaproteobacteria* (Gamma*-*) (*full line*). (D) Albumen pH. (E) Lysozyme (*dashed line*) and ovotransferrin (*full line*) concentrations.

The log copy number of the 16S rRNA gene on eggshells increased from day 1, i.e. day of clutch completion, to day 11 but this increase was not significant (r^2^ = 0.15, F_1,16_ = 2.83, P = 0.11) (Fig 1A, Table 1). In fact, eggs collected on the day of clutch completion have not been incubated yet; only eggs sampled from day 2 and onwards have been incubated. Therefore, while investigating changes in abundance from day 2 to day 11, we found that the increase in log copy number was steeper and significant (r^2^ = 0.37, F_1,8_ = 6.94, P = 0.03) (Fig 1A, Table 1). The difference between the two models was explained by a drop in abundance from day 1 to days 2–3 (t = 2.19, df = 11.3, P = 0.050).

**Table 1:** Linear mixed-effect models examining variations in bacterial abundance.

Clutch ages	Explanatory variables	*df*	*F*	*P*
(i) Day 1 to day 11	Laying order * Clutch age	1, 9	0.19	0.676
	Clutch age * Julian day	1, 14	0.87	0.367
	Julian day	1, 15	0.04	0.840
	Laying order	1, 10	1.74	0.217
	Clutch age	1, 16	2.83	0.112
(ii) Day 2 to day 11	Laying order * Clutch age	1, 6	0.38	0.562
	Clutch age * Julian day	1, 6	1.22	0.313
	Julian day	1, 8	0.007	0.938
	Laying order	1, 7	3.47	0.105
	Clutch age	1, 8	6.94	**0.030**

Two models including different clutch ages are examined: (i) from day 1 to day 11 (n = 29 eggs) and (ii) from day 2 to day 11 (n = 18 eggs). Log copy numbers of 16S rRNA gene are analyzed as estimator of bacterial abundance. Models are based on backward elimination procedure. P-values are marked up in bold when significant (P<0.05).

#### Alpha diversity metrics

Among the four α-diversity indices examined, only Shannon’s index quickly and significantly increased on eggshells in the first few days after clutch completion (up to days 3–4) and then reached a plateau while clutches got older (t = 2.43, P = 0.01) (Fig 1B). In contrast, OTU richness (t = 1.31, P = 0.19), Chao1 index (t = 1.06, P = 0.29) and Faith’s phylogenetic diversity (t = 1.19, P = 0.23) did not significantly change with clutch age (S1 Fig).

#### Phylogenetic Beta diversity

**Fig 2:**
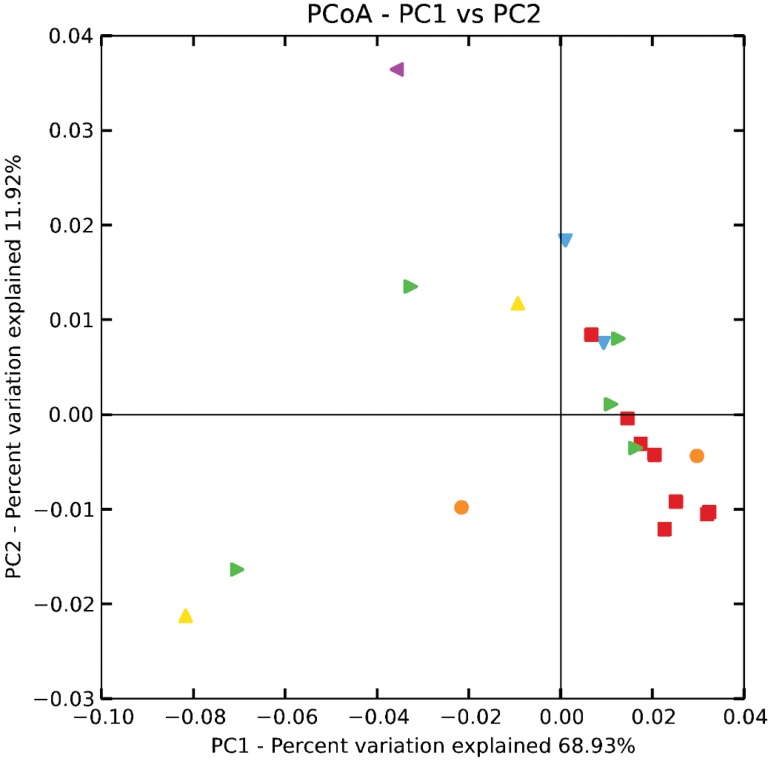
Phylogenetic β-diversity of eggshell bacterial communities at different clutch ages. Dots are plotted based on the weighted UniFrac distances among bacterial communities and visualized on a Principal Coordinates Analysis (PCoA) plot. The variability of those communities is based on the two first axes of the PCoA. The percentage of variation explained per axis is mentioned on the graph: PC1 explained 68.93% of variation among communities and PC2 explained 11.92%. Egg age is symbolized by: day 1 (*red*), 2 (*orange*), 3 (*yellow*), 5 (*green*), 8 (*blue*) and 11 (*purple*). Each dot represents the bacterial community associated with one eggshell.

While examining 1 day-old eggs, based on weighted UniFrac, we noticed their communities were phylogenetically more similar (98.0% (±0.13)) among each other, than were the ones of 5 days-old (94.2% (±0.94)). The two clutch ages significantly differed from each other (t = 4.03, df = 9.3, P = 0.003). Moreover, we observed that 1 day-old eggs preferentially clustered together along the first axis of the PCoA plot (68.9% of the variability; Fig 2). Communities of 5 days-old eggs, and more generally of other ages, were more variable and broadly distributed along the first, second (11.9% of the variability; Fig 2), and third axis (7.11% of the variability; S4 Fig) of the plots, although five of twelve eggshells overlapped with the communities of 1 day-old eggs. Differences in phylogenetic similarities between 1 day- and 5 days-old eggs were also reported for unweighted UniFrac (t = 3.06, df = 14.13, P = 0.008) and similar relationships between eggshell communities at different clutch ages were observed along the three first axes of the PCoA plots (S4 Fig).

#### Taxonomical composition of bacterial communities

At the phylum level, bacterial communities were dominated by *Proteobacteria* (95.8% ±1.21; range: 81.39–99.89%) and to a lesser extent by *Actinobacteria* (3.8% ±1.12; range: 0.11–17.45%) (S2 Fig). Although we did not observe significant variation at this taxonomical level in relation with clutch age (S2 Table), zooming in on *Proteobacteria* classes revealed significant changes. *Alphaproteobacteria* and *Gammaproteobacteria* significantly increased while clutches got older (r^2^ = 0.22, F_1,11_ = 6.63, P = 0.007; r^2^ = 0.71, F_1,12_ = 44.06, P<0.001; respectively; S2 Table). *Alphaproteobacteria* represented a small fraction of the overall communities and varied in abundance from 1.8% (±0.26; range: 1.0–3.1%) on day 1 to 2.1% (±0.26; range: 0.6–3.7%) on day 8. Likewise, *Gammaproteobacteria* increased from 6.8% (±0.95; range: 3.7–12.0%) on day 1 to 15.7% (±3.32; range: 12.4–19.0%) on day 8 (Fig 1C, S2 Fig, S1A Table). To the contrary, *Betaproteobacteria* significantly decreased with clutch age (r^2^ = 0.40, F_1,12_ = 11.52, P = 0.005; S2 Table) and were the main representative class of *Proteobacteria*, comprising 89.8% (±1.31; range: 83.1–93.6%) on day 1 and 79.3% (±3.80; range: 75.5–83.0%) on day 8 (Fig 1C, S2 Fig, S1A Table). The *Actinobacteria* phylum encompassed one single class—*Actinobacteria*—that did not change through incubation. Thus, this class remained steady, accounting for 1.4% (± 0.35; range: 0.11–3.12%) of the overall communities at day 1 and 1.8% (± 1.76; range: 0.56–3.07%) at day 8 (S2 Fig, S1A Table).

Most of the OTUs affiliated with *Alphaproteobacteria* were represented by *Ochrobacterium* and *Phyllobacterium* genera (S3A Fig). OTUs affiliated with *Gammaproteobacteria* belonged mostly to *Pseudomonas*, including *P*. *fluorescens* and *P*. *veronii*, and to a lesser extent to *Stenotrophomonas*, with both genera persisting throughout incubation (S3B Fig). *Betaproteobacteria* were dominated by one single OTU affiliated to *Herbaspirillum* sp. which comprised 91.8% of the sequences affiliated to this class (S3C Fig). Because this latter class encompassed the highest number of OTUs on eggshells, *Herbaspirillum* was the main representative genus of the overall eggshell communities. Additionally, the *Actinobacteria* class contained mainly *Rhodococcus* sp. affiliated OTUs, and a few minor species, mostly present in the first half of the incubation period (S3D Fig). Importantly, in every class, not all OTUs were detected at each incubation stage: some of them were constantly present over time while others appeared or disappeared (below detection limit) from eggshells.

### Antimicrobials in relation with clutch age

pH significantly decreased as clutches got older, from 8.1 (±0.09) at day 1 to 7.5 (±0.20) on day 11 (r^2^ = 0.28; F_1,22_ = 13.07, P = 0.002) (Fig 1D, Table 2, S1B Table). Lysozyme concentrations significantly decreased with clutch age (r^2^ = 0.24; F_1,20_ = 8.42, P = 0.009), from 1.8 mg/ml (±0.15) on day 1 to 0.9 mg/ml (±0.27) on day 8. In contrast, ovotransferrin increased over time (r^2^ = 0.24; F_1,20_ = 4.35, P = 0.049), from 9.2 mg/ml (±0.55) on day 1 to 16.1 mg/ml (±3.69) on day 8 (Fig 1E, Table 2, S1B Table).

**Table 2:** Linear mixed-effect models of albumen antimicrobial compounds.

	Explanatory variables	*df*	*F*	*P*
pH	Laying order * Clutch age	1, 16	0.003	0.956
	Julian day	1, 21	0.003	0.961
	Laying order	1, 17	1.05	0.320
	Clutch age	1, 22	13.07	**0.002**
Lysozyme concentration	Laying order * Clutch age	1, 13	3.09	0.064
(mg/ml)	Julian day	1, 19	1.24	0.278
	Laying order	1, 14	0.01	0.922
	pH	1, 14	1.35	0.264
	Clutch age	1, 20	8.42	**0.009**
Ovotransferrin concentration	Laying order * Clutch age	1, 13	0.16	0.693
(mg/ml)	Julian day	1, 19	3.08	0.096
	Laying order	1, 14	1.40	0.256
	pH	1, 14	0.52	0.484
	Clutch age	1, 20	4.35	**0.049**

Models are based on backward elimination procedure. P-values are marked up in bold when significant (P<0.05).

### Repeatability and Coefficient of Variation (CV)

**Fig 3:**
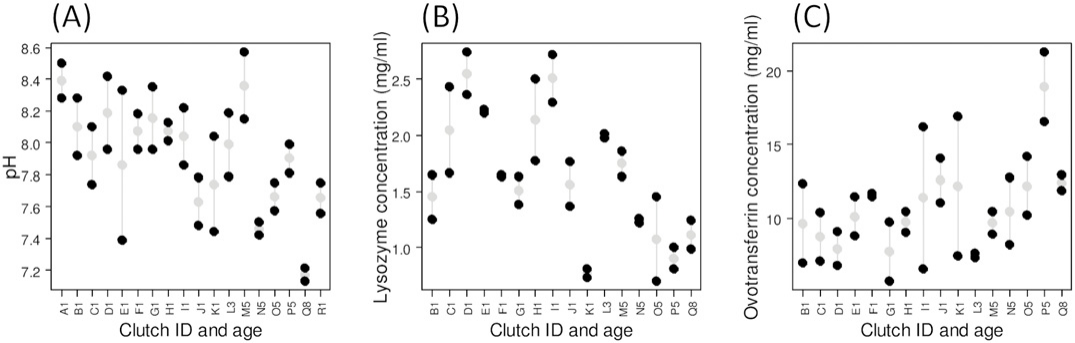
Repeatability of albumen antimicrobials among and within clutches. Clutch ID is given by a letter and followed by its age (*LetterNumber*), and is consistent across plots. Clutch age corresponds to the number of days that the complete clutch spent in the nest; day 1 is the day of clutch completion. Only complete nests (i.e containing two eggs) are plotted and ordered by clutch age. Within each plot, one clutch is represented by the values of its two eggs (*black dots*) and by their mean value (±S.E.) (*grey dots with error-bars*). Clutches are ordered by age, the youngest starting on the left part of the graphs. (A) pH, (B) lysozyme and (C) ovotransferrin concentrations.

pH did not differ between two eggs of the same clutch (χ^2^ = 1.71, P = 0.19), independent of clutch age, and repeatability was 0.31 (±0.22) (Fig 3A). As for lysozyme, two eggs from the same nest had significantly more similar concentrations compared to two eggs of two random nests (χ^2^ = 9.72, P = 0.002), independent of clutch age. Lysozyme repeatability was 0.67 (±0.14) (Fig 3B), indicating that the variation among clutches (CVa = 0.33) was relatively higher than the variation within clutches (CVw = 0.13). Ovotransferrin concentrations were not different among nests (χ^2^ = 0.05, P = 0.82), independent of clutch age. This was confirmed by a low repeatability of 0.05 (±0.25) (Fig 3C), indicating that the variation among clutches (CVa = 0.25) was similar to the variation within clutches (CVw = 0.24). Lysozyme and ovotransferrin concentrations did not correlate with each other (F_1,12_ = 0.64, P = 0.44).

### Do antimicrobials and bacterial communities correlate?

By analyzing the relationship between taxonomical composition (at the phylum—used as surrogates for Gram-positive and Gram-negative types—and class levels), or α-diversity indices with antimicrobial compounds, we found no significant correlation (Table 3). Interestingly, at the OTU level, one OTU affiliated with *Rhodococcus erythropolis* (Gram-positive) showed a negative and a positive correlation with ovotransferrin concentrations (r = -0.74, P = 0.001) and pH (r = 0.62, P = 0.008), respectively. Additionally, another OTU affiliated with *Stenotrophomonas* sp. (Gram-negative) showed a quasi-significant negative correlation with lysozyme concentrations (r = -0.49, P = 0.052) (Table 4).

**Table 3:** Linear mixed-effect models examining the relationship between antimicrobial compounds and bacterial communities.

	Explanatory variables	*df*	*F*	*P*
pH	Proteobacteria^a^ * Actinobacteria ^b^	1, 2	0.84	0.456
	Actinobacteria ^b^	1, 3	0.06	0.828
	Proteobacteria^a^	1, 4	2.84	0.167
	Gammaproteobacteria	1, 1	0.02	0.905
	Alphaproteobacteria	1, 2	0.18	0.715
	Gammaproteobacteria	1, 3	0.17	0.708
	Betaproteobacteria	1, 4	4.86	0.092
	Shannon's diversity	1, 4	3.16	0.150
	OTU richness	1, 4	3.19	0.145
	Faith's phylogenetic diversity	1, 4	3.54	0.133
	Chao 1	1, 4	1.75	0.256
Lysozyme concentration	Proteobacteria^a^ * Actinobacteria ^b^	1, 2	8.43	0.101
(mg/ml)	Proteobacteria^a^	1, 3	0.12	0.754
	Actinobacteria ^b^	1, 4	0.05	0.834
	Alphaproteobacteria	1, 1	0.40	0.642
	Betaproteobacteria	1, 2	0.57	0.529
	Actinobacteria	1, 3	0.14	0.737
	Gammaproteobacteria	1, 4	5.55	0.087
	Shannon's diversity	1, 4	1.72	0.259
	OTU richness	1, 4	0.26	0.637
	Faith's phylogenetic diversity	1, 4	0.08	0.793
	Chao 1	1, 4	0.11	0.752
Ovotransferrin concentration	Proteobacteria^a^ * Actinobacteria ^b^	1, 2	0.04	0.865
(mg/ml)	Proteobacteria^a^	1, 3	0.13	0.744
	Actinobacteria ^b^	1, 4	0.04	0.860
	Betaproteobacteria	1, 1	0.12	0.784
	Actinobacteria	1, 2	0.09	0.797
	Gammaproteobacteria	1, 3	1.25	0.345
	Alphaproteobacteria	1, 4	2.28	0.206
	Shannon's diversity	1, 4	0.03	0.958
	OTU richness	1, 4	0.004	0.953
	Faith's phylogenetic diversity	1, 4	0.55	0.498
	Chao 1	1, 4	0.26	0.635

^a^ OTUs affiliated to the *Proteobacteria* phylum are used as representative of Gram-negative bacteria.

^b^ OTUs affiliated to the *Actinobacteria* phylum are used as representative of Gram-positive bacteria.

Several bacterial community characteristics are examined such as the taxonomical composition at the phylum or class levels, and the four α-diversity indices. Sample sizes are such as 17 eggs for pH, 16 eggs for lysozyme or ovotransferrin concentrations. Lines separate each new model.

**Table 4:** Correlation between relative abundance of main eggshell OTUs and antimicrobial compounds.

OTU ID	Lysozyme		Ovotransferrin		pH		Phylum	Class	Closest hit	Accession number	Similarity (%)
	r	p-value	r	p-value	r	p-value					
Egg_Actino_14	0.01	0.982	-0.31	0.241	-0.13	0.606	Actinobacteria	Actinobacteria	*Rhodococcus erythropolis*	AJ131637	100
Egg_Actino_20	-0.05	0.856	-0.74	**0.001**	0.62	**0.008**	Actinobacteria	Actinobacteria	*Rhodococcus erythropolis*	AJ131637	100
Egg_Actino_21	-0.05	0.849	0.35	0.189	-0.32	0.204	Actinobacteria	Actinobacteria	*Propionibacterium acnes*	AB108484	100
Egg_Alpha_1	0.21	0.439	-0.34	0.197	-0.04	0.865	Proteobacteria	Alphaproteobacteria	*Phyllobacterium myrsinacearum*	AJ011330	99
Egg_Alpha_9	-0.34	0.191	0.15	0.576	-0.26	0.316	Proteobacteria	Alphaproteobacteria	*Ochrobactrum anthropi*	AY513493	100
Egg_Alpha_18	-0.32	0.223	0.11	0.683	-0.01	0.963	Proteobacteria	Alphaproteobacteria	*Ochrobactrum anthropi*	AY513493	100
Egg_Beta_4	0.09	0.739	-0.02	0.947	0.40	0.109	Proteobacteria	Betaproteobacteria	*Herbaspirillum huttiense*	DQ356897	99
Egg_Beta_5	0.21	0.424	-0.31	0.246	-0.12	0.644	Proteobacteria	Betaproteobacteria	*Ralstonia insidiosa*	AF488779	99
Egg_Beta_10	-0.07	0.791	-0.002	0.994	-0.37	0.144	Proteobacteria	Betaproteobacteria	*Rhodococcus* sp.	FJ973466	100
Egg_Beta_19	0.31	0.248	-0.24	0.379	0.34	0.188	Proteobacteria	Betaproteobacteria	*Herbaspirillum rubrisubalbicans*	AJ238356	99
Egg_Gamma_5	0.26	0.339	0.15	0.580	-0.01	0.971	Proteobacteria	Gammaproteobacteria	*Stenotrophomonas maltophilia*	AB008509	100
Egg_Gamma_7	-0.18	0.508	0.10	0.723	0.15	0.576	Proteobacteria	Gammaproteobacteria	*Pseudomonas fluorescens*	AF228366	99
Egg_Gamma_10	-0.23	0.394	0.11	0.678	0.39	0.125	Proteobacteria	Gammaproteobacteria	*Pseudomonas fluorescens*	AF228366	100
Egg_Gamma_11	-0.08	0.782	-0.11	0.686	-0.22	0.396	Proteobacteria	Gammaproteobacteria	*Pseudomonas fluorescens*	AF228366	99
Egg_Gamma_13	0.27	0.308	0.26	0.328	-0.21	0.427	Proteobacteria	Gammaproteobacteria	*Stenotrophomonas maltophilia*	AB008509	99
Egg_Gamma_24	-0.07	0.793	0.34	0.201	-0.35	0.165	Proteobacteria	Gammaproteobacteria	*Pseudomonas borealis*	AJ012712	100
Egg_Gamma_27	-0.49	0.052	0.20	0.450	-0.36	0.153	Proteobacteria	Gammaproteobacteria	*Stenotrophomonas* sp.	FJ529843	74

Correlations between antimicrobials and bacterial OTUs are done with Pearson correlation. Pearson's r value ranges from -1 (perfect negative correlation) to +1 (perfect positive correlation). The value 0 indicates no relationship. The associated P-value indicates the probability that the OTU relative abundance is correlated with the tested category (lysozyme concentrations (mg/ml), ovotransferrin concentrations (mg/ml), or pH) across eggshells. P-values <0.05 are marked up in bold. OTU ID corresponds to the bacterial class to which OTU is affiliated: ‘Alpha’ stands for *Alphaproteobacteria*, ‘Actino’ for *Actinobacteria*, ‘Beta’ for *Betaproteobacteria*, and ‘Gamma' for *Gammaproteobacteria*. Each representative sequence per OTU was compared to the Ribosomal Database Project and assigned to its closest hit, its accession number, and its percentage of similarities shared.

## Discussion

We simultaneously studied bacterial communities on eggs and antimicrobials in eggs during the entire incubation in a wild tropical passerine. Our results revealed that the bacterial communities on eggshells are dynamic in abundance, structure and composition, and that antimicrobial activities substantially change as eggs aged. Additionally, we showed that two eggs from the same clutch share equivalent amounts of antimicrobials, independent of clutch age, supporting the idea of similar immune defences within clutches. We also observed limited correlation between bacterial communities and antimicrobial compounds over time. Instead of creating a selective pressure on immunity, we suggest that certain microbes associated with eggshells may contribute to prevent infections, emphasizing the need to delve deeper into the ecological functions of the microorganisms involved.

### Dynamics of bacterial communities on eggshells and their possible protective role against external microbial invasions

After a drop between the first and the second-third days after clutch completion, once incubation started, bacterial abundance increased over time. This increase confirmed our previous findings on homing pigeon eggshells [24] and the increased abundance on mallard [21] and magpie eggshells [22] between two incubation stages, but contrasted with a study on pearly-eyed thrashers which did not find such pattern [20]. Contrasting results may arise from various factors, including methodologies, sampling techniques of bacterial cells, bird species and environmental/climatic conditions. Foremost, the comparison between two incubation stages might limit the depiction of the bacterial dynamics and lead to biased interpretations of the overall effect of incubation on bacterial abundance. Over a continuum of incubation days, we could however observe a reduction in abundance on the day of clutch completion highlighting new aspects of the eggshell microbiome dynamics. This abundance drop could be explained by large temperature changes experienced by eggs: eggs face 41–43°C in the female tract [53, 54], lower environmental temperatures in the nest, and are back up to 34–37°C as incubation starts [55, 56]. More generally, variations in nest microclimate and/or ambient environmental parameters may promote bacterial turnover as some species—likely bacteria transmitted from the reproductive/digestive female tracts to eggshells—may die, while new ones—from environmental and maternal origins—could colonize eggshells.

Eggshell bacterial community structure and composition shifted during incubation. Specifically, communities associated with freshly laid eggs were more phylogenetically clustered than those from eggs at later incubation stages. Similarly to our results, Lee *et al*. [22] found that eggshell bacterial assemblages of incubated magpie eggs became more dispersed and more dissimilar at a late incubation stage (18 days of incubation) compared with an earlier stage (3 days) (but see Grizard *et al*. [24]). Similarities among eggshell microbiomes soon after laying could be explained by the presence of cloacae-associated bacteria, transmitted from the female reproductive/digestive tracts to their eggs at laying, as shown for pied flycatchers [23, 57]. Vertical transmission paired with the fact that eggs were sampled from females breeding in the same site, likely having similar diet, thus similar gut microbiota (e.g. [58]), might contribute to the clustering of communities associated with newly laid eggs.

The taxonomical composition analysis revealed the predominance of *Proteobacteria*. Although *Alpha-*, *Gamma-*, and *Betaproteobacteria* classes changed over incubation, bacterial communities were consistently dominated by two OTUs affiliated with *Herbaspirillum* sp. and to a lesser extent to *Pseudomonas* sp. The genus *Herbaspirillum* is known to abundantly colonize poaceous plants [59] which are commonly used as lining in red-capped lark nests. Likewise, the genus *Pseudomonas* is widespread in diverse habitats including soil [60, 61], feathers [62, 63], and nests [64–66]. *Pseudomonas* were also found to abound at different incubation stages in house wren eggs [25] and pied flycatcher eggs and cloacae [23, 57]. Although the eggshell-related bacterial genera are commonly found in environmental samples, suggesting horizontal transfer of bacteria from the environment to eggshells, some of them were already present on the first day of clutch completion indicating that they can be also vertically transmitted.

The absence of *Enterobacteriaceae*, *Staphylococcaceae* and *Enterococcaceae*, families containing major egg pathogens and commonly described on eggs [20, 22, 24, 25], is striking. All together these families accounted for less than 1% of our overall communities. It would be interesting to examine whether this low proportion could indicate that they are outcompeted by the dominant bacterial species. *Herbaspirillum* and *Pseudomonas* are well known as producers of siderophores that could prevent the proliferation of other microbes [67, 68]. Experimental studies investigating the competitiveness of those two genera on potentially pathogenic species may demonstrate the protective role of the egg microbiome against external invasions due to the complexity for foreign cells to invade indigenous communities (e.g. [69]).

### Dynamics of antimicrobials during incubation and their potential relationship with microbes

Substantial changes in antimicrobial compounds occurred during incubation in red-capped lark egg albumen. For instance, pH constantly decreased towards neutrality which is consistent with the fact that as the embryo grows it produces carbon dioxide resulting in a pH decline [70]. Chicken studies however reported a peak of pH (> 9) two days after incubation began, before returning to neutrality [26, 28]. A short pH burst might create unfavorable conditions to microbial growth and boost antimicrobial activities [27]. Our results indicate that this pattern of pH variation, described in chicken eggs, might differ among bird species.

Lysozyme concentration decreased while ovotransferrin concentration increased during incubation. In line with our results, antimicrobial activities of domestic white leghorn egg albumen were also shown to vary over time. Cunningham reported an overall decrease in lysing activity through time [28], but Fang *et al*. reported its decrease only from the second day of incubation [29]. Additionally, while Cunningham established a decrease in iron-binding activity, Fang *et al*. noted its increase at early incubation [29, 71]. Changes in protein activities shared some similarities with red-capped lark eggs which may arise from embryonic growth and subsequent albumen modifications. A decrease in lysing activity may result from early lysozyme degradation soon after the onset of incubation [29] or alternatively from its physical unavailability [28] due to its binding to other proteins like ovomucin [72]. In contrast, ovotransferrin increase may occur because of water loss [73] or water shift among the egg components [29], and/or from the degradation of the vitelline membrane (separating yolk from albumen), which is rich in proteins, including ovotransferrin that would be released in albumen [71].

Antimicrobials may also vary in two other aspects: how much females invest in their first egg compared to their second one, and how much the investment differs among females. First, we found no effect of laying order on antimicrobial compounds while several earlier studies found that lysozyme decreased [30, 32] or increased [33] with laying sequence. Our findings are however consistent with a study on eight bird species which found little support for antimicrobial differences between eggs of the same clutch [15]. Previous studies hypothesized that more antimicrobials must be invested into eggs longer exposed prior to incubation because of larger infection risks (e.g. [1, 15]), but our study pointed out the quasi-absence of potential pathogenic species associated with red-capped lark eggshells. If bacterial communities efficiently prevent infections, the differential immune investment within a clutch might be minimized. Moreover, we observed that two eggs of the same clutch shared a similar amount of lysozyme and that among-clutch differences existed. As all eggs come from the same field site, and may face similar microbes, variation in antimicrobials might be caused by female age, physiology, and/or genetic factors [15, 74]. Moreover, as large climate variations may alter antimicrobial allocation [18], it would be interesting to combine the study of environmental factors with the current microbiome on eggshells, in order to strengthen the comprehension of the link between those microorganisms and egg immunity, therefore providing new insights in embryo protection.

The level of antimicrobials in eggs did not vary neither with the abundance of *Proteobacteria* (Gram-positive) or *Actinobacteria* (Gram-negative) phyla, the most abundant classes, nor the α-diversity indices. However, at the species level, lysozyme tended to correlate with one OTU assigned to *Stenotrophomonas* sp. (although not significantly—P = 0.052), and pH and ovotransferrin significantly correlated with the presence of an OTU assigned to *Rhodococcus erythropolis*. Despite limited evidence about the link between bacterial communities and antimicrobial compounds, those latter results suggest that digging deeper into the identification of bacterial species might provide a better understanding of the eventual roles these microorganisms play in the eggs. The limited evidence we observed between microbiome and antimicrobials may arise from various factors. Bacteria present on eggshells may not reflect the actual ones present inside the eggs and thus species able to colonize their contents. Extracting bacterial DNA from albumen would precisely describe the species capable of trans-shell penetration (e.g. [6]). Moreover, lysozyme and ovotransferrin might work in synergy: lysozyme was shown to potentiate ovotransferrin activity towards a particular *E*. *coli* strain [75]. Investigating antimicrobial activity of complete albumen, as recently done on chicken eggs [76], might therefore give complementary information and yield additional ecological insights. Lastly, studies sampling higher number of eggs might bring new perspectives into this relationship between eggshell microbiome and immune defences.

## Supporting Information

S1 Appendix Daily climatic data recorded over the whole year 2012. (XLSX)

S2 Appendix Sampling information and antimicrobial compound and bacterial community data on the collected eggs. (XLSX)

S1 Fig α-diversity indices in relation with the clutch age. (A) Species richness (number of OTUs) (t = 1.311, P = 0.19), (B) Chao1 richness index (t = 1.06, P = 0.29), and (C) Faith’s phylogenetic diversity (t = 1.19, P = 0.23) are reported for twenty eggshells.
(PDF)

S2 Fig Distribution of the Operational Taxonomic Units (OTUs) incorporating phyla and classes in relation with clutch age. Taxonomical lanes are ordered by clutch age, from the youngest (day1) to the oldest (day11). Each age is associated with a sample name (*Letters*, *from -a to -n*). When laying order is known, letters are associated with 1 or 2. Taxonomical phyla (*phy*) and classes (*cla*) are annotated. Only *Proteobacteria* are represented at the class level, including *Gamma*-, *Delta*-, *Beta*- and *Alphaproteobacteria*. (A) The histogram takes into account all phyla and classes. (B) The histogram is a zoom in on the first histogram excluding *Proteobacteria* classes.
(PDF)

S3 Fig Phylogenetic trees of Operational Taxonomic Units (OTUs) affiliated to four bacterial classes during incubation. (A) *Alphaproteobacteria*, (B) *Gammaproteobacteria*, (C) *Betaproteobacteria*, and (D) *Actinobacteria* affiliated OTUs are represented in separated trees. Days after clutch completion are represented by: day 1 (*red*), 2 (*orange*), 3 (*yellow*), 5 (*green*), 8 (*blue*) and 11 (*purple*). Trees are built with MEGA 5.2. Sample sequences are compared with the Ribosomal Database Project (RDP) (http://rdp.cme.msu.edu/). Trees are generated using Neighbor Joining (Bootstrap values based on 1,000 repetitions). Sequences share at least 99% of nucleotide identity. Trees (.nwk format), with their associated OTU tables (.txt format), are built using the Interactive Tree of Life (iTOL), online tool.
(PDF)

S4 Fig Principal Coordinates Analysis (PCoA) plots of the bacterial communities associated with eggshells at different clutch ages. Plots are based on weighted (A, B, C) and unweighted (D, E, F) UniFrac distance matrices. The variability of eggshell communities is based on the three first axes of the PCoA. Those three axes account for 87.96% of the variability in eggshell communities based on weighted UniFrac, and for 47.95% based on unweighted UniFrac. The percentage of variation explained per axis (PC) is mentioned on the graph. Egg age is symbolized by: day 1 (*red*), 2 (*orange*), 3 (*yellow*), 5 (*green*), 8 (*blue*) and 11 (*purple*). Each dot represents the bacterial community associated with one eggshell.
(PDF)

S1 Table Bacterial communities and antimicrobial compounds associated with red-capped lark eggs. (DOCX)

S2 Table Linear mixed-effect models of the relative abundance of bacterial phyla and classes associated with red-capped lark eggshells. Phylum and class abundances, given in percentages, are individually tested against clutch age. F tests and related P-values are reported for each model. P-values are marked up in bold when significant (P<0.05).
(DOCX)

## References

[1] Cook MI, Beissinger SR, Toranzos GA, Rodriguez RA, Arendt WJ. Trans-shell infection by pathogenic micro-organisms reduces the shelf life of non-incubated bird’s eggs: a constraint on the onset of incubation? Proc R Soc Lond. 2003;270: 2233–2240. 10.1098/rspb.2003.2508 14613609 PMC1691504

[2] Cook MI, Beissinger SR, Toranzos GA, Rodriguez RA, Arendt WJ. Microbial infection affects egg viability and incubation behavior in a tropical passerine. Behav Ecol. 2055;16: 30–36. 10.1093/beheco/arh131

[3] Cook MI, Beissinger SR, Toranzos GA, Arendt WJ. Incubation reduces microbial growth on eggshells and the opportunity for trans-shell infection. Ecol Lett. 2005;8: 532–537. 10.1111/j.1461-0248.2005.00748.x 21352457

[4] Peralta-Sánchez JM, Soler JJ, Martín-Platero AM, Knight R, Martínez-Bueno M, Møller AP. Eggshell bacterial load is related to antimicrobial properties of feathers lining barn swallow nests. Microb Ecol. 2014;67: 480–487. 10.1007/s00248-013-0338-5 24317898

[5] Soler JJ, Martín-Vivaldi M, Peralta-Sánchez JM, Ruiz-Rodríguez M. Antibiotic-producing bacteria as a possible defence of birds against pathogenic microorganisms. Open Ornithol J. 2010;3: 93–100.

[6] Javŭrková V, Albrecht T, Mrázek J, Kreisinger J. Effect of intermittent incubation and clutch covering on the probability of bacterial trans-shell infection. Ibis. 2013;156: 1–13. 10.1111/ibi.12126

[7] Beissinger SR, Cook MI, Arendt WJ. The shelf life of bird eggs: testing egg viability using a tropical climate gradient. Ecology. 2005;86: 2164–2175. 10.1890/04-1624

[8] Board RG. Properties of avian egg shells and adaptive value. Biol Rev Camb Philos Soc. 1982;57: 1–28.

[9] Mine Y, Oberl C, Kassaify Z. Eggshell matrix proteins as defense mechanism of avian eggs. J Agric Food Chem. 2003;51: 249–253. 10.1021/jf020597x 12502416

[10] Wellman-Labadie O, Picman J, Hincke MT. Antimicrobial activity of the Anseriform outer eggshell and cuticle. Comp Biochem Physio. 2008;149: 640–649. 10.1016/j.cbpb.2008.01.001 18289902

[11] Brooks J, Hale HP. The mechanical properties of the thick white of the hen’s egg. Biochim Biophys Acta. 1959;32: 237–250. 13628737 10.1016/0006-3002(59)90574-8

[12] Board RG, Fuller R. Non-specific antimicrobial defences of the avian egg, embryo and neonate. Biol Rev Camb Philos Soc. 1974;49: 15–49. 4594672 10.1111/j.1469-185x.1974.tb01297.x

[13] Board RG, Tranter HS. The microbiology of eggs In: Standelman WJ, Cotterill OJ, editors. Egg science and technology; 1995 pp. 81–104.

[14] Wellman-Labadie O, Picman J, Hincke MT. Avian antimicrobial proteins: structure, distribution and activity. World Poultry Sci J. 2007;63: 421–438. 10.1017/S0043933907001559

[15] Shawkey MD, Kosciuch KL, Liu M, Rohwer FC, Loos ER, Wang JM, et al Do birds differentially distribute antimicrobial proteins within clutches of eggs? Behav Ecol. 2008;19: 920–927. 10.1093/beheco/arn019

[16] Wellman-Labadie O, Picman J, Hincke MT. Enhanced c-type lysozyme content of wood duck (*Aix sponsa*) egg white: an adaptation to cavity nesting? Physiol Biochem Zool. 2008;81: 235–245. 10.1086/524149 18190286

[17] Horrocks NPC, Hine K, Hegemann A, Ndithia HK, Shobrak M, Williams JB, et al Are antimicrobial defences in bird eggs related to climatic conditions associated with risk of trans-shell microbial infection? Front Zool. 2014;11: 1–9. 10.1186/1742-9994-11-49 25057281 PMC4107615

[18] D’Alba L, Oborn A, Shawkey MD. Experimental evidence that keeping eggs dry is a mechanism for the antimicrobial effects of avian incubation. Naturwissenschaften. 2010;97: 1089–1095. 10.1007/s00114-010-0735-2 21057768

[19] Ruiz-De-Castañeda R, Vela AI, González-Braojos S, Briones V, Moreno J. Drying eggs to inhibit bacteria: incubation during laying in a cavity nesting passerine. Behav Process. 2011;88: 142–148. 10.1016/j.beproc.2011.08.012 21889974

[20] Shawkey MD, Firestone MK, Brodie EL, Beissinger SR. Avian incubation inhibits growth and diversification of bacterial assemblages on eggs. PloS One 2009;4: e4522 10.1371/journal.pone.0004522 19225566 PMC2639702

[21] Giraudeau M, Czirják GÁ, Duval C, Bretagnolle V, Gutierrez C, Heeb P. An experimental test in mallards (*Anas platyrhynchos*) of the effect of incubation and maternal preen oil on eggshell microbial load. J Ornithol. 2014;155: 671–677. 10.1007/s10336-014-1050-z

[22] Lee WY, Mincheol K, Jablonski PG, Choe JC, Lee SI. Effect of incubation on bacterial communities of eggshells in a temperate bird, the Eurasian magpie (*Pica pica*). Plos One 2014;9: e103959 10.1371/journal.pone.0103959 25089821 PMC4121233

[23] Ruiz-de-Castañeda R, Vela AI, Lobato E, Briones V, Moreno J. Prevalence of potentially pathogenic culturable bacteria on eggshells and in cloacae of female pied flycatchers in a temperate habitat in central Spain. J Field Ornithol. 2011;82: 215–224. 10.1111/j.1557-9263.2011.00324.x

[24] Grizard S, Dini-Andreote F, Tieleman BI, Salles JF. Dynamics of bacterial and fungal communities associated with eggshells during incubation. Ecol Evol. 2014;4: 1140–1157. 10.1002/ece3.1011 24772289 PMC3997328

[25] Potter BA, Carlson BM, Adams AE, Voss MA. An assessment of the microbial diversity present on the surface of naturally incubated house wren eggs. Open Ornithol J. 2013;6: 32–39.

[26] Romanoff AL, Romanoff A. Changes in pH of albumen and yolk in the course of embryonic development under natural and artificial incubation. Biol Bull. 1929;57: 300–306.

[27] Tranter HS, Board RG. The influence of incubation temperature and pH on the antimicrobial properties of hen egg albumen. J Appl Bacteriol. 1984;56: 53–61. 6706888 10.1111/j.1365-2672.1984.tb04696.x

[28] Cunningham FE. Changes in egg white during incubation of the fertile egg. Poult Sci. 1974;53: 1561–1565. 4850959 10.3382/ps.0531561

[29] Fang J, Ma M, Jin Y, Qiu N, Ren G, Huang X, et al Changes in the antimicrobial potential of egg albumen during the early stages of incubation and its impact on the growth and virulence response of *Salmonella* Enteritidis. Ital J Anim Sci. 2012;11: 92–97. 10.4081/ijas.2012.e17

[30] Saino N, Dall’ara P, Martinelli R, Møller AP. Early maternal effects and antibacterial immune factors in the eggs, nestlings and adults of the barn swallow. J Evol Biol. 2002;15: 735–743. 10.1046/j.1420-9101.2002.00448.x

[31] Cucco M, Grenna M, Pellegrino I, Malacarne G. Egg-sequence rather than mating preference influences female egg investment in the red-legged partridge. Ethol Ecol Evol. 2011;23: 343–357. 10.1080/03949370.2011.584565

[32] Cucco M, Guasco B, Malacarne G, Ottonelli R. Effects of beta-carotene on adult immune condition and antibacterial activity in the eggs of the grey partridge, *Perdix perdix* . Comparative Biochem Physiol. 2007;147: 1038–1046. 10.1016/j.cbpa.2007.03.014 17462926

[33] Bonisoli-Alquati A, Rubolini D, Romano M, Cucco M, Fasola M, Caprioli M, et al Egg antimicrobials, embryo sex and chick phenotype in the yellow-legged gull. Behav Ecol Sociobiol. 2010;64: 845–855. 10.1007/s00265-010-0901-8

[34] Godard RD, Wilson CM, Frick JW, Siegel PB, Bowers BB. The effects of exposure and microbes on hatchability of eggs in open-cup and cavity nests. J Avian Biol. 2007;38: 709–716. 10.1111/j.2007.0908-8857.04052.x

[35] Peralta-Sánchez JM, Martín-Vivaldi M, Martín-Platero AM, Martínez-Bueno M, Oñate M, Ruiz-Rodríguez M, et al Avian life history traits influence eggshell bacterial loads: a comparative analysis. Ibis. 2012;154:725–737. 10.1111/j.1474-919X.2012.01256.x

[36] Horrocks NPC, Hegemann A, Matson KD, Hine K, Jaquier S, Shobrak M, et al Immune indexes of larks from desert and temperate regions show weak associations with life history but stronger links to environmental variation in microbial abundance. Physiol Biochem Zool. 2012;85: 504–515. 10.1086/666988 22902379

[37] Del Hoyo J, Elliott A, Christie DA. Handbook of birds of the world: contigas to pipits and wagtails. Vol. 9 BirdLife International: Lynx Edicions Barcelona; 2004

[38] Hamburger V, Hamilton HL. A series of normal stages in the development of chick embryo. J Morphol. 1951;8: 49–92. 24539719

[39] Ricklefs RE, Starck J. Embryonic growth and development In: Starck JM, Ricklefs RE, editors. Avian growth and development—Evolution within the altricial-precocial spectrum; 1998 pp. 31–58.

[40] Caporaso JG, Kuczynski J, Stombaugh J, Bittinger K, Bushman FD, Costello EK, et al QIIME allows analysis of high-throughput community sequencing data. Nat Methods. 2010;7: 335–336. 10.1038/nmeth0510-335 20383131 PMC3156573

[41] DeSantis TZ, Hugenholtz P, Larsen N, Rojas M, Brodie EL, Keller K, et al Greengenes, a chimera-checked 16S rRNA gene database and workbench compatible with ARB. Appl Environ Microbiol. 2006;72: 5069–5072. 10.1128/AEM.03006-05 16820507 PMC1489311

[42] Edgar RC. Search and clustering orders of magnitude faster than BLAST. Bioinformatics. 2010;26: 2460–2461. 10.1093/bioinformatics/btq461 20709691

[43] Caporaso JG, Bittinger K, Bushman FD, DeSantis TZ, Andersen GL, Knight R. PyNAST: a flexible tool for aligning sequences to a template alignment. Bioinformatics. 2010;26: 266–267. 10.1093/bioinformatics/btp636 19914921 PMC2804299

[44] Wang Q, Garrity GM, Tiedje JM, Cole JR. Naive Bayesian classifier for rapid assignment of rRNA sequences into the new bacterial taxonomy. Appl Environ Microbiol. 2007;73: 5261–5267. 10.1128/AEM.00062-07 17586664 PMC1950982

[45] Lozupone CA, Lladser ME, Knights D, Stombaugh J, Knight R. UniFrac: an effective distance metric for microbial community comparison. ISME J. 2011;5: 169–172. 10.1038/ismej.2010.133 20827291 PMC3105689

[46] Tamura K, Peterson D, Peterson N, Stecher G, Nei M, Kumar S. MEGA5: molecular evolutionary genetics analysis using maximum likelihood, evolutionary distance, and maximum parsimony methods. Mol Biol Evol. 2011;28: 2731–2739. 10.1093/molbev/msr121 21546353 PMC3203626

[47] Letunic I, Bork P. Interactive Tree Of Life (iTOL): an online tool for phylogenetic tree display and annotation. Bioinformatics. 2007;23: 127–128. 10.1093/bioinformatics/btl529 17050570

[48] Horrocks NPC, Tieleman BI, Matson KD. A simple assay for measurement of ovotransferrin—a marker of inflammation and infection in birds. Methods Ecol Evo. 2011;2: 518–526. 10.1111/j.2041-210X.2011.00096.x

[49] Pinheiro J, Bates D, DebRoy S, Sarkar D. nlme: linear and nonlinear mixed effects models. R package version 3.1–101. R Development Core Team. 2011. Available: http://cran.r-project.org/web/packages/nlme/index.html.

[50] Bates D, Maechler M, Bolker B, Walker S. lme4: linear mixed-effects models using eigen and S4. R package version 1.0–4. R Development Core Team. 2013. Available: http://cran.r-project.org/web/packages/lme4/index.html

[51] Versteegh MA, Helm B, Dingemanse NJ, Tieleman BI. Repeatability and individual correlates of basal metabolic rate and total evaporative water loss in birds: a case study in European stonechats. Comp Biochem Physiol A. 2008;150: 452–457. 10.1016/j.cbpa.2008.05.006 18571446

[52] *R Development Core Team*. R: A language and environment for statistical computing. R Foundation for Statistical Computing, Vienna, Austria; 2013 10.3758/s13428-013-0330-5

[53] Prinzinger R, Preβmar A, Schleucher E. Body temperature in birds. Comp Biochem Physiol. 1991;4: 499–506.

[54] Clarke A, Rothery P. Scaling of body temperature in mammals and birds. Funct Ecol. 2007;22: 58–67. 10.1111/j.1365-2435.2007.01341.x

[55] Tieleman BI, Williams JB, Ricklefs RE. Nest attentiveness and egg temperature do not explain the variation in incubation periods in tropical birds. Funct Ecol. 2004;18: 571–577.

[56] Wang JM, Weathers WW. Egg laying, egg temperature, attentiveness, and incubation in the western bluebird. Wilson J Ornithol. 2009;121: 512–520.

[57] Ruiz-de-Castañeda R, Vela AI, Lobato E, Briones V, Moreno J. Prevalence of *Salmonella* and *Yersinia* in free-living pied flycatchers (*Ficedula hypoleuca*) in central Spain. J Zoo Wildl Med. 2011;42: 313–316. 10.1638/2010-0056.1 22946412

[58] Lozupone CA, Stombaugh JI, Gordon JI, Jansson JK, Knight R. Diversity, stability and resilience of the human gut microbiota. Nature. 2012;489: 220–230. 10.1038/nature11550 22972295 PMC3577372

[59] Monteiro RA, Balsanelli E, Wassem R, Marin AM, Brusamarello-Santos LCC, Schmidt MA, et al *Herbaspirillum*-plant interactions: microscopical, histological and molecular aspects. Plant Soil. 2012;356: 175–196. 10.1007/s11104-012-1125-7

[60] Berg G, Eberl L, Hartmann A. The rhizosphere as a reservoir for opportunistic human pathogenic bacteria. Environ Microbiol. 2005;7: 1673–1685. 10.1111/j.1462-2920.2005.00891.x 16232283

[61] Garbeva P, Postma J, van Veen JA, van Elsas JD. Effect of above-ground plant species on soil microbial community structure and its impact on suppression of *Rhizoctonia solani AG3* . Environ Microbiol. 2006;8: 233–246. 10.1111/j.1462-2920.2005.00888.x 16423012

[62] Shawkey MD, Mills KL, Dale C, Hill GE. Microbial diversity of wild bird feathers revealed through culture-based and culture-independent techniques. Microb Ecol. 2005;50: 40–47. 10.1007/s00248-004-0089-4 16132422

[63] Bisson I, Marra PP, Burtt EH Jr, Sikaroodi M, Gillevet PM. A molecular comparison of plumage and soil bacteria across biogeographic, ecological, and taxonomical scales. Microb Ecol. 2007;54: 65–81. 10.1007/s00248-006-9173-2 17334855

[64] Xin YH, Zhang DC, Liu HC, Zhou HL, Zhou YG. *Pseudomonas tuomuerensis* sp. nov., isolated from a bird’s nest. Int J Syst Evol Micr. 2009;59: 139–143. 10.1099/ijs.0.000547-0 19126738

[65] Goodenough AE, Stallwood B. Intraspecific variation and interspecific differences in the bacterial and fungal assemblages of blue tit (*Cyanistes caeruleus*) and great tit (*Parus major*) nests. Microb Ecol. 2010;59: 221–232. 10.1007/s00248-009-9591-z 19830477

[66] Goodenough AE, Stallwood B. Differences in culturable microbial communities in bird nestboxes according to orientation and influences on offspring quality in great tits (*Parus major*). Microb Ecol. 2012;63: 986–995. 10.1007/s00248-011-9992-7 22183046

[67] Rosconi F, Davyt D, Martínez V, Martínez M, Abin-Carriquiry JA, Zane H, et al Identification and structural characterization of serobactins, a suite of lipopeptide siderophores produced by the grass endophyte *Herbaspirillum seropedicae* . Environ Microbiol. 2013;15: 916–927. 10.1111/1462-2920.12075 23320867

[68] Saha R, Saha N, Donofrio RS, Bestervelt LL. Microbial siderophores: a mini review. J Basic Microbiol. 2013;53:303–317. 10.1002/jobm.201100552 22733623

[69] He X, McLean JS, Guo L, Lux R, Shi W. The social structure of microbial community involved in colonization resistance. ISME J. 2014;8: 564–574. 10.1038/ismej.2013.172 24088624 PMC3930314

[70] Reijrink IAM, Meijerhof R, Kemp B, van den Brand H. The chicken embryo and its micro environment during egg storage and early incubation. World Poultry Sci J. 2008;64: 581–598. 10.1017/S0043933908000214

[71] Fang J, Ma M, Jin Y, Qiu N, Huang Q, Sun S, et al Liquefaction of albumen during the early incubational stages of the avian embryo and its impact on the antimicrobial activity of albumen. J Food Agric Environ. 2012;10: 423–427.

[72] Kato A, Imoto T, Yagishita K. The binding groups in ovomucin-lysozyme interaction. Agri Biol Chem. 1975;39: 541–544.

[73] Ar A, Rahn H. Water in the avian egg: overall budget of incubation. Am Zool. 1980;20: 373–384.

[74] Saino N, Romano M, Ambrosini R, Ferrari RP, Møller AP. Timing of reproduction and egg quality covary with temperature in the insectivorous barn swallow, *Hirundo rustica* . Funct Ecol. 2004;18: 50–57. 10.1046/j.0269-8463.2004.00808.x

[75] Ko KY, Mendoncam AF, Ismail H, Ahn DU. Ethylenediaminetetraacetate and lysozyme improves antimicrobial activities of ovotransferrin against *Escherichia coli* O157:H7. Poult Sci. 2009;88: 406–414. 10.3382/ps.2008-00218 19151356

[76] Bedrani L, Helloin E, Guyot N, Réhault-Godbert S, Nys Y. Passive maternal exposure to environmental microbes selectively modulates the innate defences of chicken egg white by increasing some of its antibacterial activities. BMC Microbiol. 2013;13:1–13. 10.1186/1471-2180-13-128 23758641 PMC3681677

