# Supplementary figures and images for: Shifts in Bacterial Communities of Eggshells and Antimicrobial Activities in Eggs during Incubation in a Ground-Nesting Passerine
Source: PLoS One. 2015 Apr 16;10(4):e0121716. doi: 10.1371/journal.pone.0121716 (PMC4400097; doi:10.1371/journal.pone.0121716)

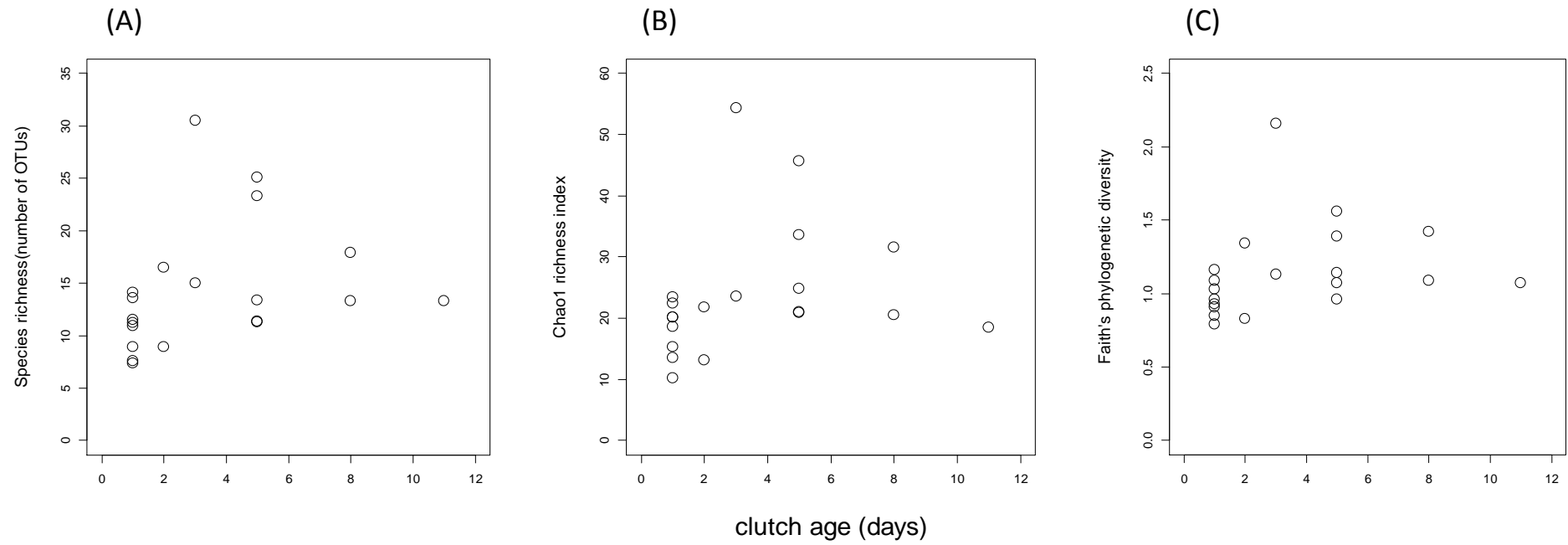

**Figure S1:  $\alpha$ -diversity indices in relation with the clutch age.**

Supplement: S1 Fig — (A) Species richness (number of OTUs) (t = 1.311, P = 0.19), (B) Chao1 richness index (t = 1.06, P = 0.29), and (C) Faith’s phylogenetic diversity (t = 1.19, P = 0.23) are reported for twenty eggshells. (PDF) [file pone.0121716.s003.pdf]
