# Supplementary material for: Shifts in Bacterial Communities of Eggshells and Antimicrobial Activities in Eggs during Incubation in a Ground-Nesting Passerine
Source: PLoS One. 2015 Apr 16;10(4):e0121716. doi: 10.1371/journal.pone.0121716 (PMC4400097; doi:10.1371/journal.pone.0121716)
Supplement: S2 Fig — Taxonomical lanes are ordered by clutch age, from the youngest (day1) to the oldest (day11). Each age is associated with a sample name (Letters, from -a to -n). When laying order is known, letters are associated with 1 or 2. Taxonomical phyla (phy) and classes (cla) are annotated. Only Proteobacteria are represented at the class level, including Gamma-, Delta-, Beta- and Alphaproteobacteria. (A) The histogram takes into account all phyla and classes. (B) The histogram is a zoom in on the first histogram excluding Proteobacteria classes. (PDF) [file pone.0121716.s004.pdf]

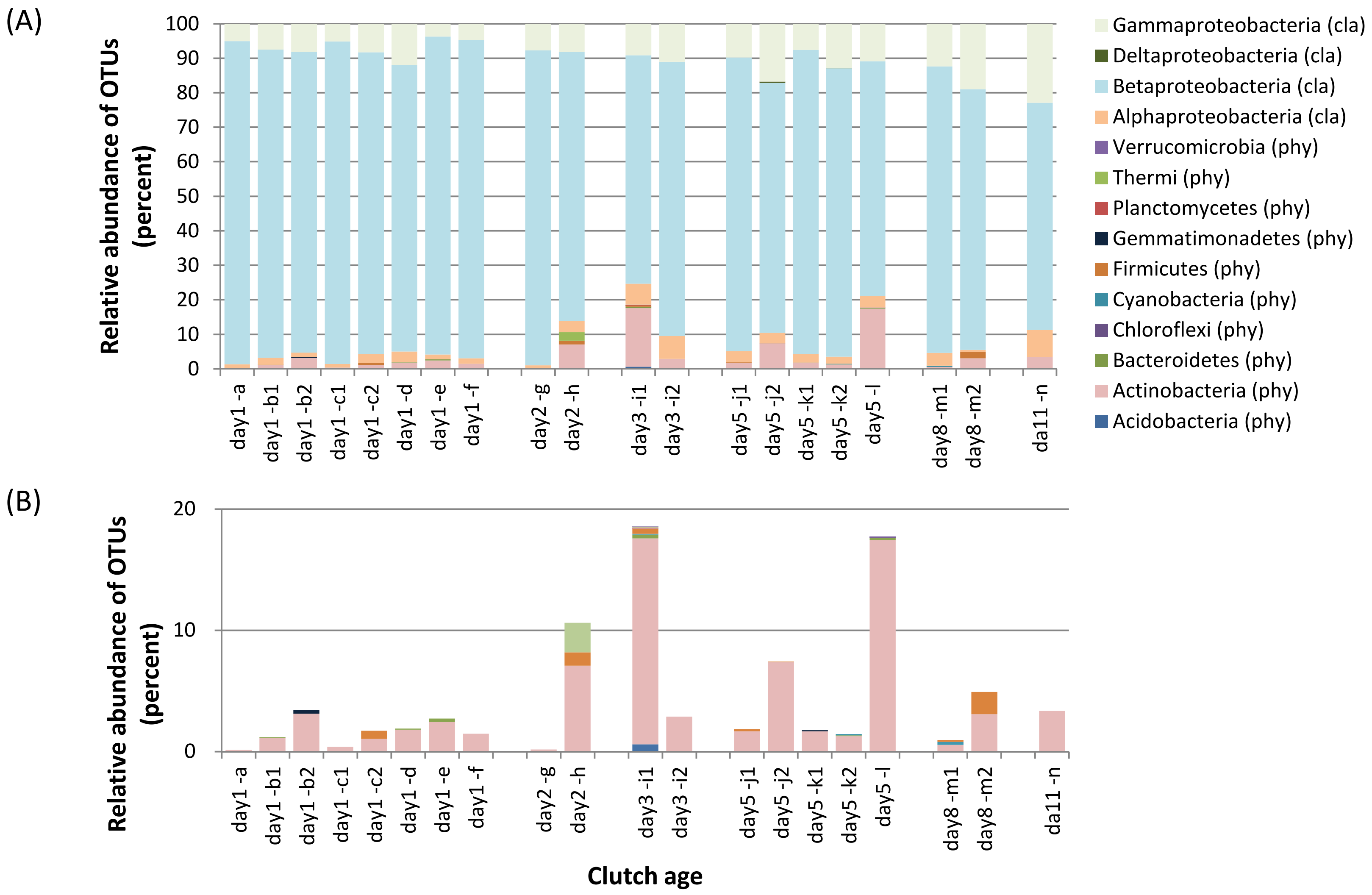

Figure S2: Distribution of the Operational Taxonomic Units (OTUs) incorporating phyla and classes in relation with clutch age.
