# Supplementary material for: Shifts in Bacterial Communities of Eggshells and Antimicrobial Activities in Eggs during Incubation in a Ground-Nesting Passerine
Source: PLoS One. 2015 Apr 16;10(4):e0121716. doi: 10.1371/journal.pone.0121716 (PMC4400097; doi:10.1371/journal.pone.0121716)

Figure S3: Phylogenetic trees of Operational Taxonomic Units (OTUs) affiliated to four bacterial classes during incubation.

(A) Alphaproteobacteria

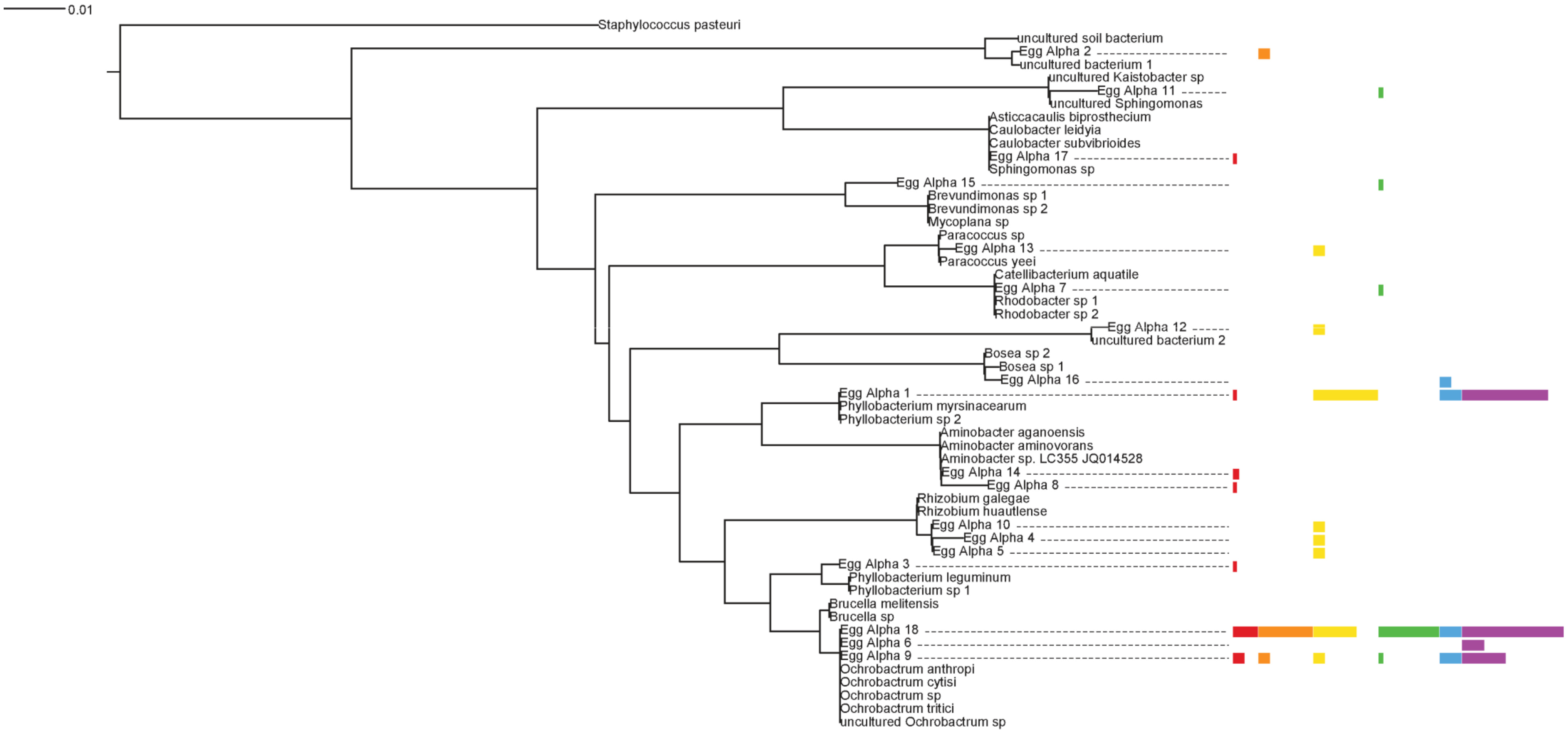

(B) Gammaproteobacteria

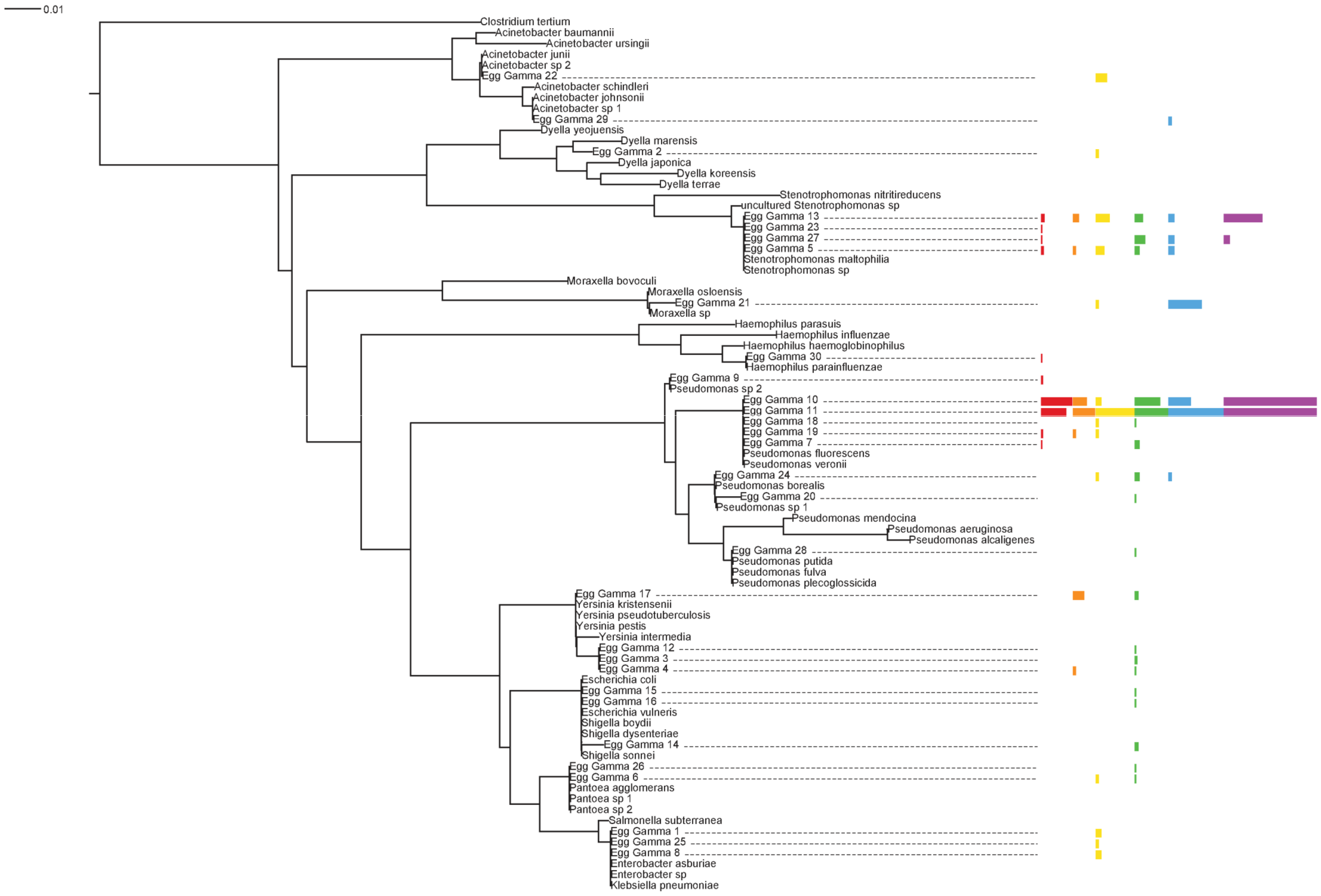

(C) Betaproteobacteria

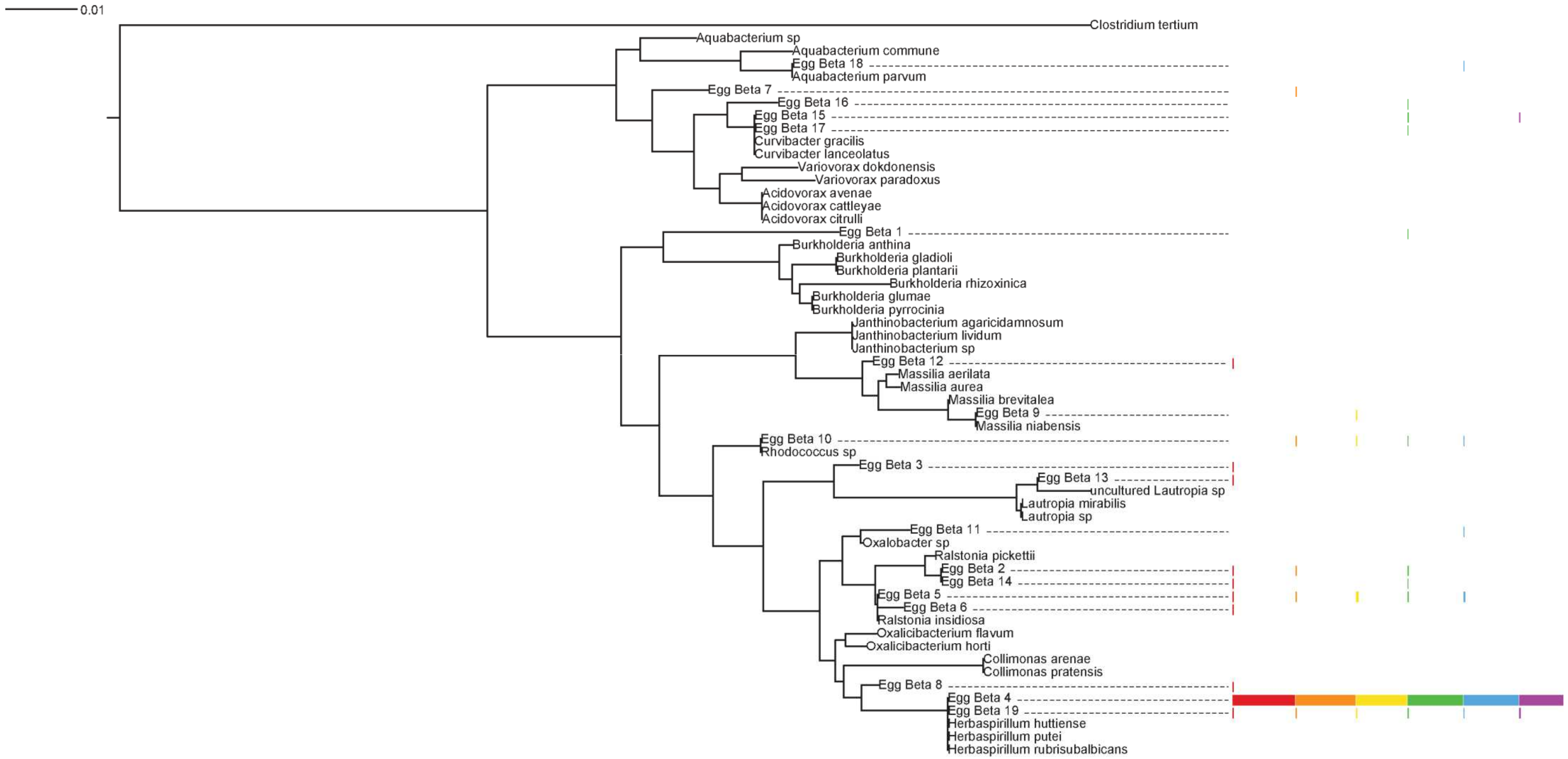

(D) Actinobacteria

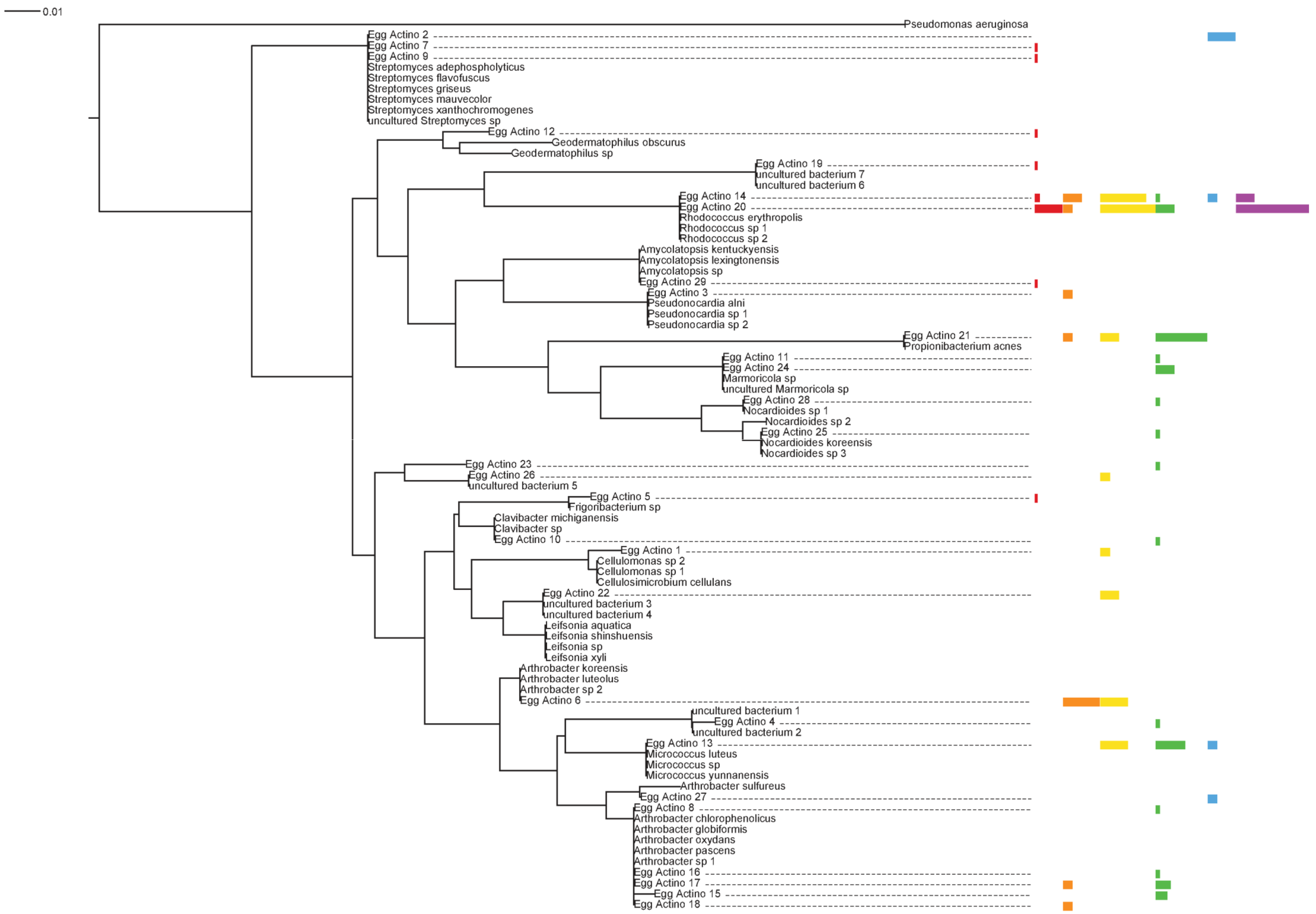

Supplement: S3 Fig — (A) Alphaproteobacteria, (B) Gammaproteobacteria, (C) Betaproteobacteria, and (D) Actinobacteria affiliated OTUs are represented in separated trees. Days after clutch completion are represented by: day 1 (red), 2 (orange), 3 (yellow), 5 (green), 8 (blue) and 11 (purple). Trees are built with MEGA 5.2. Sample sequences are compared with the Ribosomal Database Project (RDP) (http://rdp.cme.msu.edu/). Trees are generated using Neighbor Joining (Bootstrap values based on 1,000 repetitions). Sequences share at least 99% of nucleotide identity. Trees (.nwk format), with their associated OTU tables (.txt format), are built using the Interactive Tree of Life (iTOL), online tool. (PDF) [file pone.0121716.s005.pdf]
