# Supplementary material for: Shifts in Bacterial Communities of Eggshells and Antimicrobial Activities in Eggs during Incubation in a Ground-Nesting Passerine
Source: PLoS One. 2015 Apr 16;10(4):e0121716. doi: 10.1371/journal.pone.0121716 (PMC4400097; doi:10.1371/journal.pone.0121716)
Supplement: S4 Fig — Plots are based on weighted (A, B, C) and unweighted (D, E, F) UniFrac distance matrices. The variability of eggshell communities is based on the three first axes of the PCoA. Those three axes account for 87.96% of the variability in eggshell communities based on weighted UniFrac, and for 47.95% based on unweighted UniFrac. The percentage of variation explained per axis (PC) is mentioned on the graph. Egg age is symbolized by: day 1 (red), 2 (orange), 3 (yellow), 5 (green), 8 (blue) and 11 (purple). Each dot represents the bacterial community associated with one eggshell. (PDF) [file pone.0121716.s006.pdf]

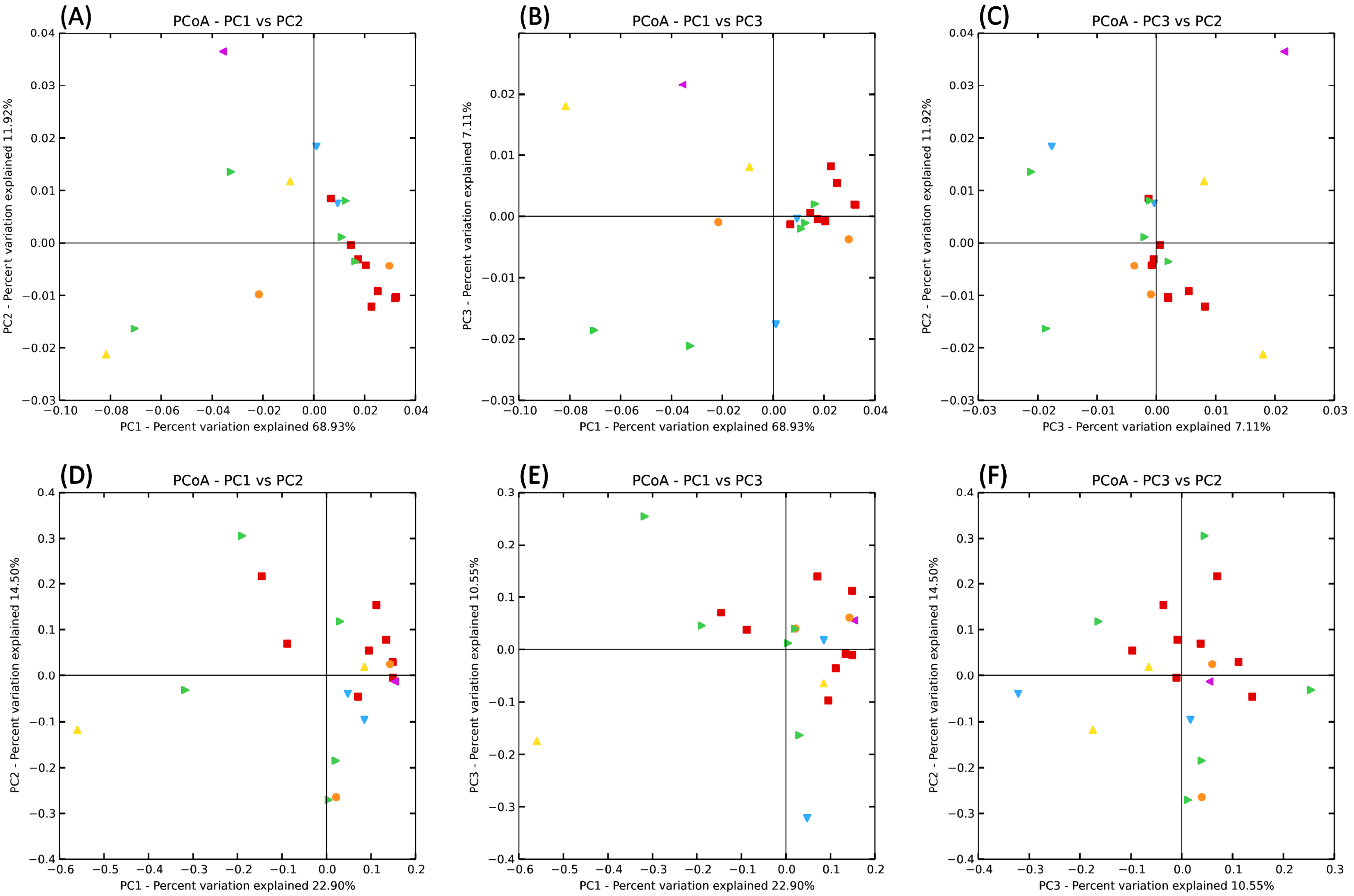

Figure S4: Principal Coordinates Analysis (PCoA) plots of the bacterial communities associated with eggshells at different clutch ages.
