# Supplementary material for: Shifts in Bacterial Communities of Eggshells and Antimicrobial Activities in Eggs during Incubation in a Ground-Nesting Passerine
Source: PLoS One. 2015 Apr 16;10(4):e0121716. doi: 10.1371/journal.pone.0121716 (PMC4400097; doi:10.1371/journal.pone.0121716)
Supplement: S1 Table — (DOCX) [file pone.0121716.s007.docx]

**Table S1: Bacterial communities and antimicrobial compounds associated with red-capped lark eggs.**

| (A) Bacterial communities |  | **Clutch age** ^a^ | | | | | |
| --- | --- | --- | --- | --- | --- | --- | --- |
|  |  | 1 | 2 | 3 | 5 | 8 | 11 |
| Abundance | log copy number of 16S rRNA gene | 3.9 (±0.19) | 3.3 (±0.40) | 3.0 (±0.11) | 4.0 (±0.40) | 4.3 (±0.77) | 5.2 (±0.09) |
|  | n nests | 7 | 3 | 1 | 4 | 1 | 1 |
|  | n eggs | 11 | 5 | 2 | 7 | 2 | 2 |
| Taxonomy (%) | Proteobacteria (phylum) | 98.4 (±0.39) | 94.6 (±5.23) | 89.3 (±7.87) | 94.0 (±3.13) | 97.1 (±1.98) | 96.7 (NA) |
|  | Actinobacteria (phylum/class) | 1.4 (±0.35) | 3.6 (±3.45) | 9.9 (±7.06) | 5.9 (±3.10) | 1.8 (±1.26) | 3.3 (NA) |
|  | Alphapreoteobacteria (class) | 1.8 (±0.26) | 2.0 (±1.23) | 6.4 (±0.28) | 2.8 (±0.23) | 2.1 (±0.26) | 8.0 (NA) |
|  | Gammaproteobacteria (class) | 6.8 (±0.95) | 8.0 (±0.21) | 10.11 (±0.95) | 11.6 (±1.56) | 15.7 (±3.32) | 22.9 (NA) |
|  | Betaproteobacteria (class) | 89.8 (±1.31) | 84.6 (±6.67) | 72.8 (±6.64) | 79.5 (±3.90) | 79.3 (±3.80) | 65.8 (NA) |
| Alpha-diversity indices | Shannon's diversity index | 0.9 (0.11) | 1.2 (0.40) | 2.1 (0.46) | 1.5 (0.29) | 1.5 (20.24) | 1.9 (NA) |
|  | Species richness (number of OTU) | 10.7 (0.90) | 12.7 (3.80) | 22.8 (7.75) | 16.9 (3.02) | 15.6 (2.30) | 13.3 (NA) |
|  | Chao 1 richness index | 18.0 (1.61) | 17.5 (4.37) | 38.9 (15.39) | 29.2 (4.71) | 26.0 (5.50) | 18.4 (NA) |
|  | Faith's phylogenetic diversity index | 1.0 (0.04) | 1.1 (0.26) | 1.6 (0.52) | 1.2 (0.11) | 1.3 (0.17) | 1.1 (NA) |
|  | n nests ^b^ | 6 | 2 | 1 | 3 | 1 | 1 |
|  | n eggs ^b^ | 8 | 2 | 2 | 5 | 2 | 1 |

| (B) Antimicrobial compounds |  | **Clutch age** ^a^ | | | | | |
| --- | --- | --- | --- | --- | --- | --- | --- |
|  |  | 1 | 2 | 3 | 5 | 8 | 11 |
| pH | values | 8.1 (±0.09) | 7.9 (±0.10) | 8.0 (±0.20) | 7.8 (±0.12) | 7.3 (±0.13) | 7.5 (±0.20) |
|  | n nests | 9 | 4 | 1 | 6 | 2 | 2 |
|  | n eggs | 16 | 8 | 2 | 10 | 3 | 3 |
| Lysozyme | concentrations (mg/ml) | 1.8 (±0.15) | 1.7 (±0.26) | 2.00 (±0.02) | 1.3 (±0.16) | 0.9 (±0.27) | - |
|  | n nests | 9 | 4 | 1 | 6 | 2 | - |
|  | n eggs | 15 | 8 | 2 | 10 | 3 | - |
| Ovotransferrin | concentrations (mg/ml) | 9.2 (±0.55) | 11.5 (±1.38) | 7.5 (±0.15) | 12.4 (±1.42) | 16.1 (±3.69) | 15.7 (NA) |
|  | n nests | 8 | 4 | 1 | 6 | 2 | 1 |
|  | n eggs | 14 | 8 | 2 | 10 | 3 | 1 |

^a^ C*lutch age* corresponds to the number of days after clutch completion.

^b^ *n nests* and *n eggs* are the same for *Taxonomy* and *Alpha-diversity indices*
