# Supplementary material for: Shifts in Bacterial Communities of Eggshells and Antimicrobial Activities in Eggs during Incubation in a Ground-Nesting Passerine
Source: PLoS One. 2015 Apr 16;10(4):e0121716. doi: 10.1371/journal.pone.0121716 (PMC4400097; doi:10.1371/journal.pone.0121716)
Supplement: S2 Table — Phylum and class abundances, given in percentages, are individually tested against clutch age. F tests and related P-values are reported for each model. P-values are marked up in bold when significant (P<0.05). (DOCX) [file pone.0121716.s008.docx]

**Table S2: Linear mixed-effect models of the relative abundance of bacterial phyla and classes associated with red-capped lark eggshells.**

|  | Explanatory variables | *df* | F | P |
| --- | --- | --- | --- | --- |
| Proteobacteria (phylum) | Laying order * Clutch age | 1, 4 | 0.29 | 0.621 |
|  | Laying order | 1, 5 | 0.15 | 0.713 |
|  | Julian day | 1, 11 | 1.43 | 0.257 |
|  | Clutch age | 1, 12 | 0.29 | 0.599 |
| Actinobacteria (phylum/class*) | Laying order * Clutch age | 1, 4 | 0.24 | 0.648 |
|  | Laying order | 1, 5 | 0.36 | 0.573 |
|  | Julian day | 1, 11 | 1.23 | 0.291 |
|  | Clutch age | 1, 12 | 0.33 | 0.576 |
| Alphaproteobacteria (class) | Laying order * Clutch age | 1, 4 | 2.95 | 0.161 |
|  | Laying order | 1, 5 | 0.12 | 0.748 |
|  | Julian day | 1, 11 | 8.83 | 0.013 |
|  | Clutch age | 1, 11 | 10.72 | **0.007** |
| Betaproteobacteria (class) | Laying order * Clutch age | 1, 4 | 0.11 | 0.754 |
|  | Laying order | 1, 5 | 0.39 | 0.559 |
|  | Julian day | 1, 11 | 3.32 | 0.096 |
|  | Clutch age | 1, 12 | 11.53 | **0.005** |
| Gammaproteobacteria (class) | Laying order * Clutch age | 1, 4 | 1.92 | 0.221 |
|  | Laying order | 1, 5 | 6.53 | 0.051 |
|  | Julian day | 1, 11 | 0.26 | 0.622 |
|  | Clutch age | 1, 12 | 44.06 | **<0.001** |

** Actinobacteria* phylum was represented by one single class (*Actinobacteria*) explaining its assignation to both phylum and class.
